# Spherical harmonics analysis reveals cell shape-fate relationships in zebrafish lateral line neuromasts

**DOI:** 10.1242/dev.202251

**Published:** 2024-01-26

**Authors:** Madeleine N. Hewitt, Iván A. Cruz, David W. Raible

**Affiliations:** ^1^ Molecular and Cellular Biology Graduate Program, University of Washington School of Medicine, Seattle, WA 98195, USA; ^2^ Department of Otolaryngology-HNS, University of Washington School of Medicine, Seattle, WA 98195, USA; ^3^ Department of Biological Structure, University of Washington School of Medicine, Seattle, WA 98195, USA

**Keywords:** Hair cell, Lateral line, Zebrafish

## Abstract

Cell shape is a powerful readout of cell state, fate and function. We describe a custom workflow to perform semi-automated, 3D cell and nucleus segmentation, and spherical harmonics and principal components analysis to distill cell and nuclear shape variation into discrete biologically meaningful parameters. We apply these methods to analyze shape in the neuromast cells of the zebrafish lateral line system, finding that shapes vary with cell location and identity. The distinction between hair cells and support cells accounted for much of the variation, which allowed us to train classifiers to predict cell identity from shape features. Using transgenic markers for support cell subpopulations, we found that subtypes had different shapes from each other. To investigate how loss of a neuromast cell type altered cell shape distributions, we examined *atoh1a* mutants that lack hair cells. We found that mutant neuromasts lacked the cell shape phenotype associated with hair cells, but did not exhibit a mutant-specific cell shape. Our results demonstrate the utility of using 3D cell shape features to characterize, compare and classify cells in a living developing organism.

## Teaser

**Summary:** Spherical harmonics analysis of 3D cell shapes allows quantitative characterization, comparison and classification of cell types and cell fate in a living developing organism.

## INTRODUCTION

Cell shape emerges from a complex interplay between fate, function and external forces (Chan et al., 2017). Quantifying cell shape during development has provided mechanistic insight into morphogenetic processes (Perez-Vale and Peifer, 2020), cell division and death (Chen et al., 1997), as well as differentiation (Watt et al., 1988; Kilian et al., 2010). However, most studies of cell shape *in vivo* have used descriptive features (such as volume and sphericity), which may not capture subtle or multifactorial variations in shape between cell types, especially in tissues with complex 3D organization. More complete 2D shape representations have been used to study cell motility and migration (Pincus and Theriot, 2007; Keren et al., 2008) but these methods cannot be easily generalized to 3D shapes. To fully appreciate the diversity of cell shape states during development and understand the relationship between cell shape and fate, robust 3D cell shape analysis methods must be applied to living developing organisms.

Recently, several 3D cell shape representations have shown promising results. One approach uses spherical harmonics (SH), where the surface of each cell is mapped to a sphere, and the SH transform is used to generate a list of coefficients that describe the shape of the cell (Ruan and Murphy, 2019). Principal components analysis (PCA) can then be applied to reduce dimensionality and facilitate interpretation of cell shape variation within and between cell populations. SH and PCA has been used to understand variation in cell and nuclear shape of IPS cells in culture (Viana et al., 2023), and to describe organ shape trajectories in murine limb and heart development (Dalmasso et al., 2022), and 3D shapes in medical imaging (Gerig et al., 2001). These methods maintain enough information to accurately reconstruct 3D cell shapes, enabling sophisticated data-driven analysis. Here we test the idea that we can use SH coefficients to cluster and classify cell types.

The zebrafish lateral line system is an ideal model for studying the relationship between cell shape, location and fate in a living developing animal. Organs of the lateral line, known as neuromasts, are small, tightly packed epithelial rosettes with apical-basal polarity, radially organized cell types and mirror symmetry (Thomas et al., 2015). Mechanosensory hair cells (HCs) are located in the center of the neuromast and surrounded by nonsensory support cells (SCs). SCs can be distinguished based on their locations, gene expression patterns and propensity for HC replacement during regeneration, and have been identified in live animals using established knock-in lines (Lush et al., 2019; Thomas and Raible, 2019). The superficial location of neuromasts and optical transparency of zebrafish larvae facilitate high resolution live imaging.

In this study, we developed a workflow to segment neuromast cells and nuclei, and analyze their 3D shapes using SH and PCA. We model cell and nucleus shape using just eight cell shape and four nucleus shape principal components (PCs). We found that HCs were clearly distinct from SCs in shape space, whereas SCs appeared to vary continuously in shape. Unsupervised clustering revealed that markers for SC subpopulations were enriched within certain clusters, suggesting these cell types have distinct cell shape features. Analysis of mutants for *atoh1a*, encoding a conserved transcription factor necessary for HC differentiation (Bermingham et al., 1999; Millimaki et al., 2007), revealed the loss of the HC shape cluster and expansion of other cell shape clusters. We also successfully built classifiers to predict HC identity from cell and nucleus shape features. Our work demonstrates that cell shape parameters can be used to characterize and classify cell types in neuromasts, laying the foundation for future studies of form, fate and function in sensory epithelia.

## RESULTS

### A semi-automated workflow accurately segments zebrafish lateral line cells and nuclei

**Fig. 1.**
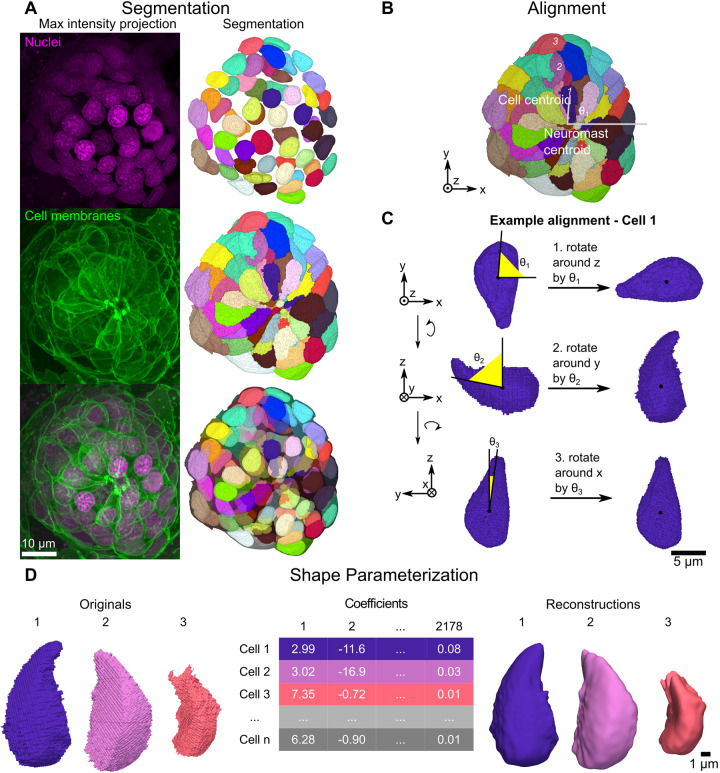
**Overview of segmentation and shape parameterization.** (A) Representative maximum intensity projections (left column) and corresponding 3D projections (right column) for nuclei labeled with DRAQ5 dye (top row), cells labeled by Tg(-8.0cldnb:LY-EGFP) (middle row) and both channels together (bottom row). (B) Image depicting how the first rotation angle is calculated. Example given for the maximum intensity projection segmentation channel of the neuromast shown in A. Cells 1, 2 and 3 are used in C and D. (C) Example of the alignment procedure as applied to cell 1 from B. To correct for radial symmetry, cells are first rotated around the *z*-axis. Cells were then rotated around the *y*-axis to correct for apicobasal tilt, and then around the *x*-axis to correct for off-parallel tilt during imaging. (D) Overview of shape parameterization using SH, as applied to cells 1, 2, and 3 from B. SH expansion is applied to original 3D surface meshes (left) to generate the cell-by-SHE coefficients table (middle), which can then be used to generate 3D reconstructions (right).

Neuromasts are epithelial organs with tightly packed, apically constricted cells that present challenges for imaging and segmentation (Fig. 1A). For all experiments, we used the zebrafish *Tg(cldnb:LY-EGFP)* transgenic line that labels neuromast cell membranes (Haas and Gilmour, 2006). In addition, some zebrafish contained knock-in alleles that labeled cell populations of interest, for analysis described below. For the numbers of neuromasts and cells from each line, refer to  Table S1. All fish were stained with the far-red nuclear dye DRAQ5 (Smith et al., 1999). Fish were imaged live at 5 days post-fertilization (dpf), when neuromasts are functionally mature.

We trained machine learning models to detect cell boundaries from membrane GFP signal and nuclei masks from DRAQ5 signal, and used model results as the foundation for instance segmentation. We first applied a distance transform watershed algorithm to segment every nucleus. Labeled nuclei were then used as seeds for marker-based watershed of membrane boundary predictions. The accuracy of segmentation was further improved through proofreading and manual correction. This semi-automated workflow produced good quality segmentations for most cells (Fig. 1A). In total, 3274 cells from 49 different neuromasts across six different imaging sessions were segmented.

### Spherical harmonics accurately represent most neuromast cell shapes

To quantitatively compare cell shapes, we used a method based on SH expansion (Viana et al., 2023). The result is a vector of coefficients that describe cell shape (organized into a cell-by-coefficients table), on which the inverse transform can be applied to create a reconstruction of the cell (Fig. 1D). To minimize variation due to cell orientation so that we could query intrinsic cell shape, we corrected for (1) the radial symmetry of cells around the apical-basal axis of the neuromast, (2) differential tilt along the radial axis as cells converge at the apical end of the rosette, and (3) tilt introduced in image acquisition resulting in the *z*-axis being slightly unparallel to the apical-basal axis. An example of a cell aligned by this procedure is shown in Fig. 1B,C.

After alignment, we applied SH expansion to generate 2178 SH coefficients for each cell (Fig. 1D). To measure reconstruction fidelity, we used directed Hausdorff distance. We found a bimodal distribution of error, with 84% of cells accurately reconstructed (Fig. S1A). Inaccurately reconstructed cells tended to be located midway between the center and periphery of the neuromast (Fig. S1B) and had shapes that rendered them unsuitable for SH expansion, such as high concavity (Fig. S1C). These cells tended to have greater cell surface area and height (Fig. S1D, top). However, nuclei of excluded cells had similar dimensions to those of retained cells (Fig. S1D, bottom). In summary, some tall, curved cells located in a ring around the middle of the neuromast were not reconstructed well by SH (421 cells, representing 16% of the original dataset), and were excluded from subsequent cell shape analysis.

### Principal components analysis reveals variations in cell shape between neuromast cell types

**Fig. 2.**
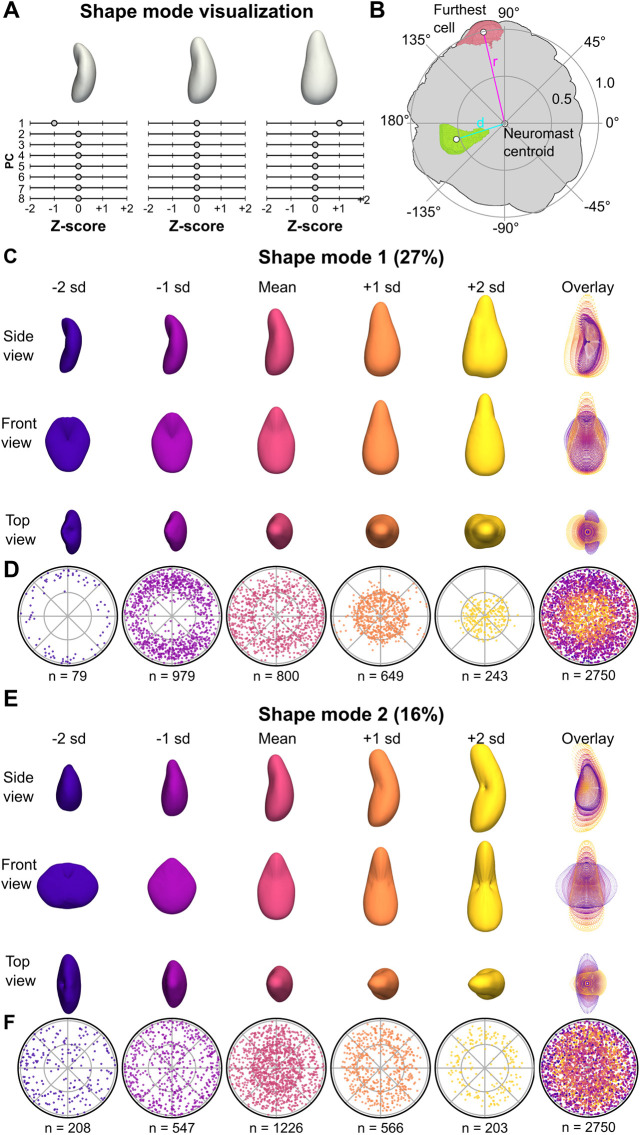
**Visualization of cell shape modes 1 and 2.** (A) Schematic depicting how shape variation was visualized. PCs were first *z*-scored to generate shape modes. Visual representations of each shape mode were generated by finding the mean cell (a hypothetical cell with a value of 0 for all 8 shape modes) and varying one shape mode (here, shape mode 1) by up to 2 standard deviations (s.d.) while holding other shape modes at the mean (0). (B) Schematic depicting how relative cell locations within each neuromast were calculated. The distance between the neuromast centroid and the furthest cell (r) in the *xy* plane was calculated. The distances between the neuromast centroid and cell centroids were divided by (r) to yield the normalized distance from the neuromast center (d). The angular location of each cell was determined in reference to the positive *x*-axis centered on the neuromast centroid. Cell locations were projected onto a unit circle with radius 1. (C) Visualization of CSM1, generated as described in A. 3D surface renderings are shown from three different views, as well as an overlay of point representations for each view (rightmost column). (D) Polar plots of relative cell locations (generated as shown in B) binned by CSM1 scores. Bins were centered on the corresponding *z*-score and included cells with values within 0.5. For the −2 s.d. and 2 s.d. plots, the upper bounds were not limited to within 0.5 (e.g. the first plot includes all cells with CSM1 values less than −1.5 s.d.). An overlay of the first five plots is shown in the rightmost column. (E) Visualization of CSM2. 3D surface renderings are shown from three different views, as well as an overlay of point representations for each view (rightmost column). (F) Polar plots of relative cell locations binned by CSM2 scores. An overlay of the first five plots is shown in the rightmost column.

To identify the main axes of shape variation, we applied principal component (PC) analysis. The 2178 SH coefficients were reduced to eight PCs representing ∼70% of the total variance (Fig. S2). A key advantage of our approach is that from any point in this eight-dimensional shape space, it is possible to calculate the corresponding SH coefficients and create 3D representations of real or theoretical cells. We wondered whether variation in shape between cell types would be continuous (indicating smooth transitions between different shapes) or discrete (suggesting some populations have features that set them more clearly apart from other cells). To visualize shape variation, each PC was first individually z-scored to generate ‘shape modes’. We used the point corresponding to the origin in shape space (where the value of all eight cell shape modes is 0) to create a visual representation of the mean cell shape. We then varied the value of one shape mode while keeping other shape modes constant (Fig. 2A). To determine how each shape mode varied with position across neuromasts, we classified cell position by corresponding shape mode value (Fig. 2B). A small number of interpretable parameters therefore captures the majority of cell shape variation.

To test whether HCs would be quantitatively distinct from SCs, we manually annotated HCs in our dataset using the presence of apical stereocilia, which are visible in the *Tg(cldnb:LY-EGFP)* transgenic line. We found that cell shape mode 1 (CSM1, representing 27% of total variance) appeared to represent ‘HC-ness’. Positive CSM1 scores corresponded with greater cell volume and width (Fig. S3B), as well as a more convex, flask-like shape (Fig. 2C). Cells with positive CSM1 scores also tended to be closer to the neuromast center (Fig. 2D; Fig. S3D), consistent with the characteristic HC location. This result suggests that much of cell shape variation in neuromasts is due to differences between HCs and SCs.

In contrast to CSM1, cell shape mode 2 (CSM2; 16% of total variance) did not distinguish between HCs and SCs; rather it appeared to distinguish among SC types. Cells with increasing CSM2 scores were progressively taller and less deep (Fig. 2E; Fig. S3B). CSM2 was also negatively associated with being located toward the neuromast periphery, particularly for SCs (Fig. 2F; Fig. S3D). HCs tended to have average CSM2 scores, clustered around zero and showed a narrower distribution than SCs (Fig. S3C). Taken together, the relationships between cell type and cell position likely explain the ‘bullseye’ pattern observed when CSM2 scores are plotted by relative cell location: SCs have higher CSM2 scores as they become more central, whereas HCs (which are also centrally located) have CSM2 scores close to zero (Fig. 2F).These observations suggest that CSM2 represents shape variation between SCs in a way that is distinct from the variation between SCs and HCs.

To further query the relationships between shape modes and cell type, we compared shape modes to geometric features and location parameters (Fig. S3). We correlated CSM1 and CSM2 to cell width, depth, height, volume, surface area and cell distance from the neuromast center (Fig. S3B,D). We found a positive correlation between CSM1 and cell volume and cell width, and a negative correlation with distance from the center of the neuromast. For CSM2, there was a positive correlation with cell height and a negative correlation with cell depth and distance from center. HCs consistently segregated with CSM1 but not CSM2 (Fig. S3C,D). Beyond CSM1 and CSM2, other shape modes appeared to represent more subtle ways in which neuromast cell shape varied (Fig. S2).

### Nuclear shape variation is captured by SHE and PCA

We also performed SHE and PCA on nucleus shape. Nuclei were aligned to account for position within the neuromast using a series of rotations similar to (but independent of) those used to align cells. Because nuclei had a much lower reconstruction error compared with cells (Fig. S1A), we did not to exclude any nuclei from analysis. Nuclear SHE coefficients were reduced to four PCs representing ∼76% of the total variance in nuclear shape (Fig. S4). As for cell shape analysis, nuclear shape PCs were z-scored to describe them as nuclear shape modes.

**Fig. 3.**
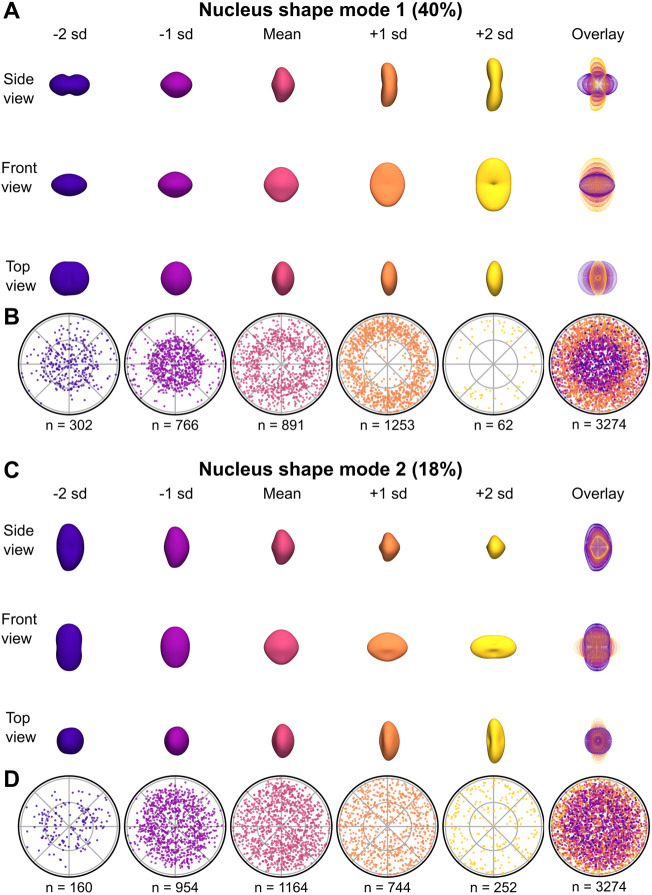
**Visualization of nuclear shape modes 1 and 2.** (A) Visualization of NSM1, generated as in Fig. 2A. 3D surface renderings are shown from three different views, as well as an overlay of point representations for each view (rightmost column). (B) Polar plots of relative cell locations (generated as in Fig. 2D) binned by NSM1 scores. An overlay of the first five plots is shown in the rightmost column. (C) Visualization of NSM2. 3D surface renderings are shown from three different views, as well as an overlay of point representations for each view (rightmost column). (D) Polar plots of relative cell locations binned by NSM2 scores. An overlay of the first five plots is shown in the rightmost column.

Nucleus shape mode 1 (NSM1, representing 40% of total variance, Fig. 3A) and nucleus shape mode 2 (NSM2, representing 18% of total variance, Fig. 3C) captured distinct aspects of variation in nuclear shape. Increasing NSM1 scores were associated with a shift from short wide shapes to a tall crescent-like morphology (Fig. 3A). NSM2 appeared to describe a progression from tall and cylindrical to flattened rectangular shapes (Fig. 3C). Nucleus shape modes 3 and 4 are summarized in Fig. S4. Comparing with other nuclear shape parameters (Fig. S5), we found NSM1 was negatively correlated with nucleus width and positively correlated with nucleus height (Fig. S5B,D). NSM2 was positively correlated with nucleus depth and negatively correlated with nucleus volume (Fig. S5B,D).

We asked to what degree nuclear shape modes distinguished between HCs and SCs. We found that NSM1 had a bimodal distribution (Fig. S5C) and was correlated with distance from the neuromast center (Fig. 3B; Fig. S5D). Like CSM1, NSM1 also appeared to distinguish between HCs and SCs, but to a lesser degree (Fig. S5C). NSM2 showed a slightly skewed distribution and a correlation with distance to the neuromast center (Fig. 3D;  Fig. S5D), with some distinction between HCs and SCs. Nuclear shape modes therefore also captured variation between HCs and SCs, although this information was represented by multiple shape modes.

### Unsupervised clustering of neuromast cells in shape space identifies groups of cells with similar shapes and locations

**Fig. 4.**
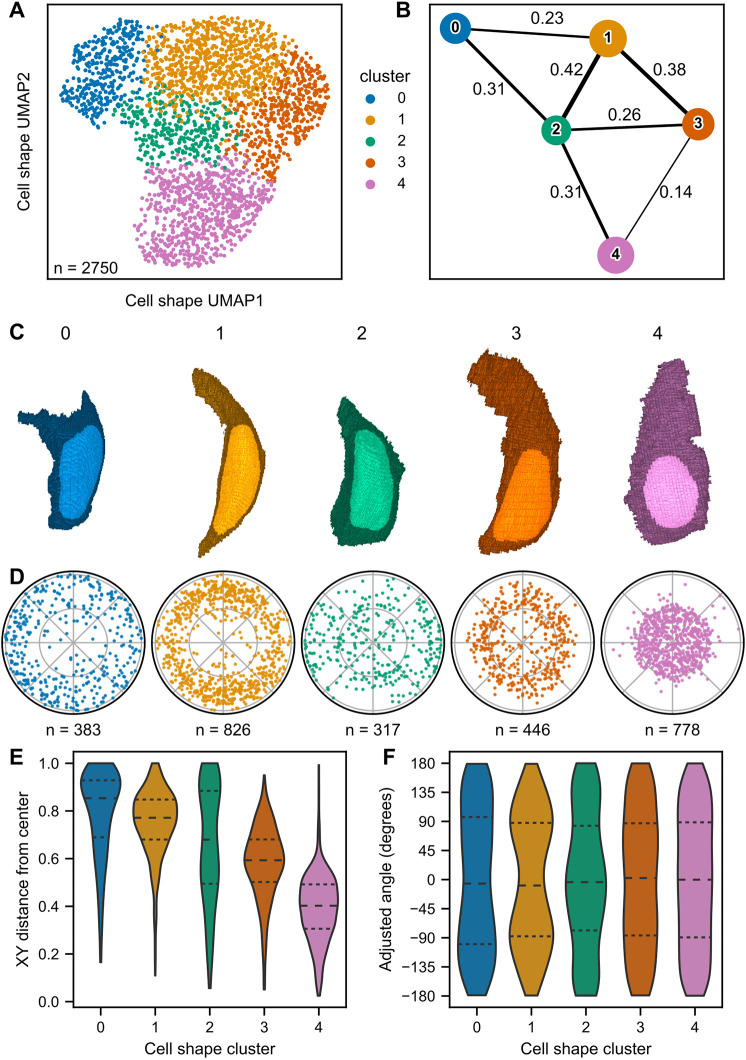
**Unsupervised clustering of cells in shape space.** (A) UMAP of neuromast cells, color coded by cell shape cluster. Colors and associated cluster numbers are used in B-F. (B) PAGA plot of cell shape clusters. Each node represents a cluster. Thickness of the line drawn between nodes indicates estimated connectivity between clusters (the value of which is shown adjacent to the corresponding line). Edges with connectivity below 0.1 are not depicted. (C) 3D projections of representative cells for each cluster, defined as the cell closest to the cluster centroid in eight-dimensional PC space. (D) Polar plots of relative neuromast locations of cells within each cluster. (E) Distributions of the cell distance from the neuromast center for each cluster. Dashed lines indicate quartiles. (F) Distributions of neuromast cell angles for each cluster. Dashed lines indicate quartiles. Angles were calculated with reference to the positive *x*-axis extending from the neuromast centroid. Cluster 1 shows a bias towards the dorsoventral poles (90° and −90°), whereas cluster 2 shows a bias to the anterior-posterior compartments (0°, 180° and −180°).

We next determined whether shape features could be used to assign neuromast cells to biologically meaningful categories beyond HCs and SCs. We performed unsupervised clustering on the first eight cell shape PCs using the Phenograph package and Leiden community detection algorithm (Levine et al., 2015; Traag et al., 2019). Five identified clusters were represented in two dimensions by a uniform manifold approximation and projection (UMAP; McInnes et al., 2018; Fig. 4A), where each cell is a point colored by cluster label. The fraction of cells within each cluster was proportional across all individual neuromasts (Fig. S6D), suggesting that clustering is influenced by shared shape parameters and not due to differences between neuromasts. To quantify the relationships between clusters, we performed partition-based graph abstraction (PAGA; Wolf et al., 2019). Relative strength of association is represented by line thickness (Fig. 4B). PAGA analysis revealed greater similarities between clusters 0/2, 1/2/3 and 2/4.

To visualize the characteristic shapes of cells within each cluster, we identified the cell closest to the centroid of each cluster in eight-dimensional PC space as a ‘representative cell’ (Fig. 4C). Cells in clusters 0 tended to be shorter with greater depth, whereas cells in cluster 3 tended to be tall and wide. Cells in clusters 1 and 2 appeared to have intermediate shapes. Cells in cluster 4 tended to have flask shapes reminiscent of HCs; quantifying manually annotated HCs in each cluster confirmed that most HCs were assigned to cluster 4 (Fig. S6E) and cluster 4 was primarily composed of HCs (Fig. S6F).

Cells from a given cluster tended to be found at distinct radial distances from the neuromast center (Fig. 4D,E), and we used this order to assign cluster number labels. However, we also found that some clusters were distributed in distinct quadrants of the neuromast (Fig. 4D,F). This difference in distribution was particularly apparent for cluster 1, where cells were more concentrated within the dorsoventral quadrants (Fig. 4F, represented by adjusted angles 90° and −90°), and cluster 2, where cells were more often found within the anteroposterior quadrants (Fig. 4F, represented by an adjusted angle of 0°). These results suggest that grouping neuromast cells purely using shape descriptors, without explicitly including information about cell type or identity, recapitulates known aspects of neuromast organization (e.g. concentric and radially symmetric cell populations).

**Fig. 5.**
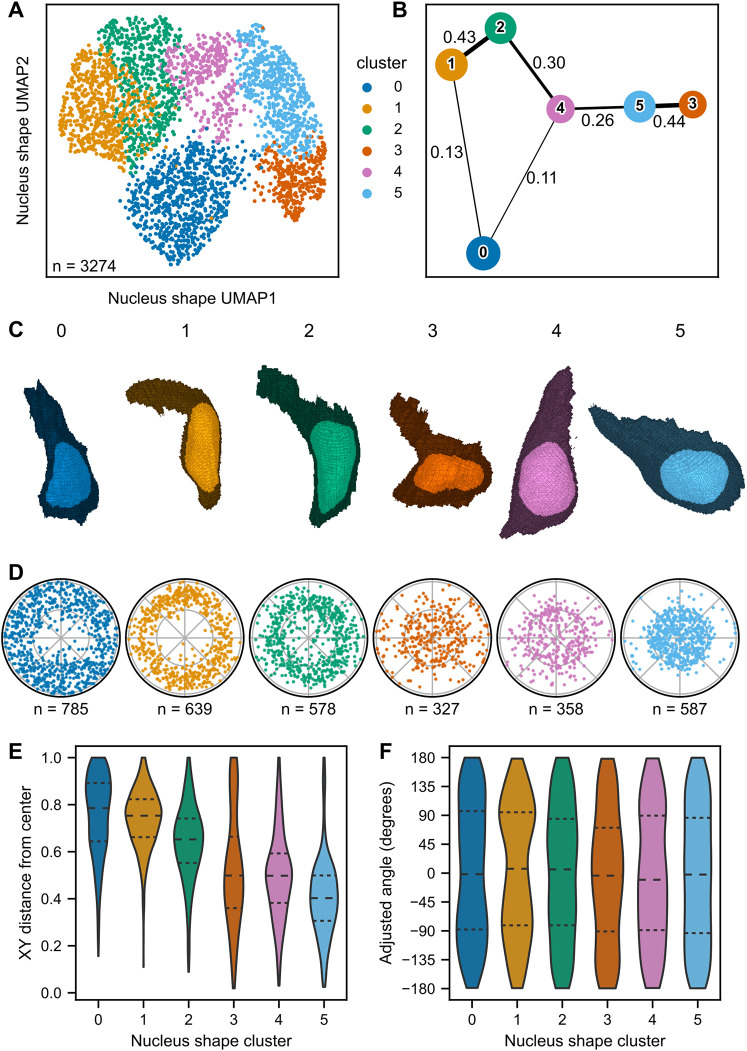
**Unsupervised clustering of nuclei in shape space.** (A) UMAP of neuromast cells, color coded by nucleus shape cluster. Colors and associated cluster numbers are used in B-F. (B) PAGA plot of nucleus shape clusters. Each node represents a cluster. The thickness of the line drawn between nodes indicates the estimated connectivity between clusters (the value of which is shown adjacent to the corresponding line). Edges with connectivity below 0.1 are not depicted. (C) 3D projections of representative cells for each nuclear cluster, defined as the cell closest to the cluster centroid in four-dimensional PC space. The original segmentation masks are depicted (not SHE reconstructions). (D) Polar plots of relative locations of cells within each cluster. (E) Distributions of the cell distance from the neuromast center for each cluster. Dashed lines indicate quartiles. (F) Distributions of neuromast cell angles for each cluster. Dashed lines indicate quartiles. Angles were calculated with reference to the positive *x*-axis extending from the neuromast centroid.

We also performed unsupervised clustering on the first four nucleus shape PCs. Similar to cell shape clusters, we found distinct nucleus shape clusters (Fig. 5A) whose relative relationships could be measured through PAGA analysis (Fig. 5B). As for cells, we identified representative nuclei for each cluster (Fig. 5C). Clusters 4 and 5 were primarily composed of HCs, and cluster 1 was enriched for dorsoventral cells (Fig. 5D). Nucleus shape clusters exhibited distinct spatial locations within the neuromast (Fig. 5D-F). However, nucleus shape clusters did not appear to distinguish as well between SC subpopulations compared with cell shape clusters. As the nucleus shape dataset contains cells that were excluded from cell shape analysis, we looked at the distribution of excluded cells across nucleus shape clusters (as a way to understand whether a particular class of cells had been excluded). We found excluded cells in all five nucleus shape clusters, suggesting that excluded cells can have a full range of nucleus shape phenotypes (Fig. S1E). Based on these results, we decided to perform the remaining analysis using only cell shape clusters.

### Cell fate markers of SC subpopulations are associated with distinct cell shape phenotypes

**Fig. 6.**
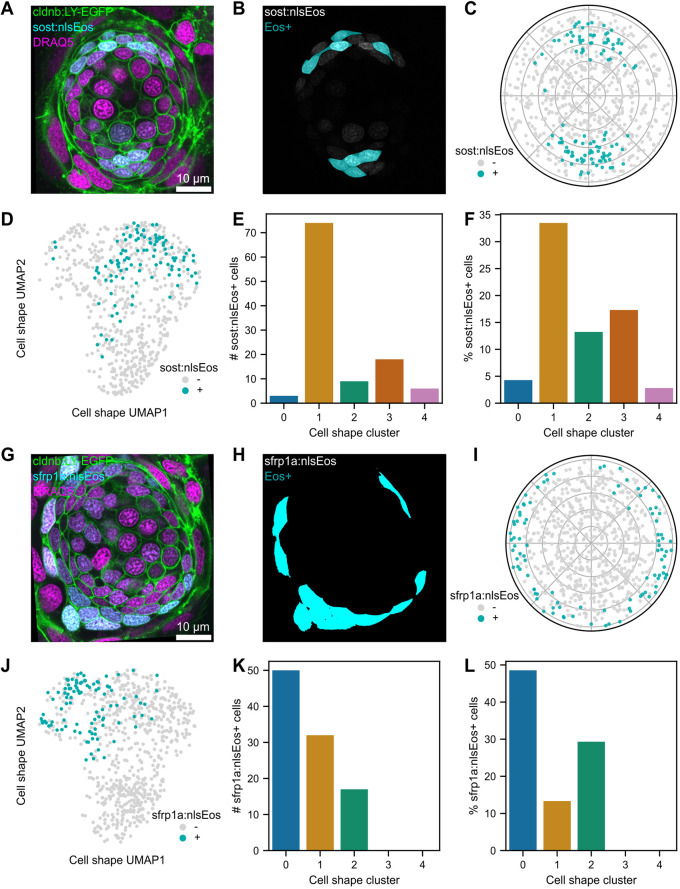
**Location and shape characteristics of cells expressing *sost:NLS-Eos* and *sfrp1a:NLS-Eos*.** (A) Single confocal slice of a neuromast from *sost:NLS-Eos; Tg(-8.0cldnb:LY-EGFP)* fish stained with DRAQ5. Image contrast was adjusted for visibility. (B) The same slice as in A, with the *sost:NLS-Eos* channel (gray) overlaid with a mask indicating cells classified as Eos^+^ (cyan). (C) Polar plot showing relative cell locations of locations of Eos^+^ (cyan) and Eos^−^ (gray) cells. (D) UMAP of Eos^+^ (cyan) and Eos^−^ (gray) cells from *sost:NLS-Eos*. (E) *sost:NLS-Eos*^+^ cells plotted as raw counts within each cell shape cluster. (F) *sost:NLS-Eos*^+^ cells plotted as the percentage of each cluster. (G) Single confocal slice of a neuromast from *sfrp1a:NLS-Eos; Tg(-8.0cldnb:LY-EGFP)* fish stained with DRAQ5. Image contrast was adjusted for visibility. (H) The same slice as in A, with the *sfrp1a:NLS-Eos* channel (gray) overlaid with a mask indicating cells classified as Eos^+^ (cyan). (I) Polar plot showing relative cell locations of locations of Eos^+^ (cyan) and Eos^−^ (gray) cells. (J) UMAP of Eos^+^ (cyan) and Eos^−^ (gray) cells from *sfrp1a:NLS-Eos*. (K) *sfrp1a:NLS-Eos*^+^ cells plotted as raw counts within each cell shape cluster. (L) *sfrp1a:NLS-Eos*^+^ cells plotted as the percentage of each cluster.

SC subpopulations have characteristic transcriptional profiles and behaviors during regeneration: DV cells act as HC progenitors while peripheral cells can replenish DV cells depleted by HC regeneration (Lush et al., 2019; Thomas and Raible, 2019). We therefore examined neuromasts from knock-in lines that mark these distinct SC subpopulations. The *sost:NLS-Eos* line marks cells in the DV compartment of the neuromast (Fig. 6A-C). We measured the mean intensities of Eos fluorescence in each cell, normalizing to z-score for each individual neuromast. We then classified cells as Eos^+^ if they had fluorescence with z-score greater than 1 (Fig. 6B, Fig. S6A). An example neuromast with positively labeled cells is shown in Fig. 6B. As expected, *sost:NLS-Eos*^+^ cells were found in DV quadrants (Fig. 6B,C). We next asked whether *sost:NLS-Eos*^+^ cells were differentially distributed across clusters (Fig. 6E,F). Most *sost:NLS-Eos^+^* cells were found in cluster 1 (Fig. 6E). 33% of cluster 1 cells were s*ost:NLS-Eos*^+^, the highest proportion of any cluster (Fig. 6F). Some *sost:NLS-Eos^+^* cells were also found in clusters 2 and 3, making up 13% and 17% of clusters 2 and 3, respectively (Fig. 6F).

The *sfrp1a:NLS-Eos* line marks peripheral SCs (Fig. 6G-I). We used the same threshold method to classify cells as Eos^+^ (Fig. 6H, Fig. S6B). Eos^+^ cells were found in the neuromast periphery (Fig. 6H,I). Most *sfrp1a:NLS-Eos^+^* cells were found within cluster 0, making up about half of the cells in this cluster (Fig. 6K,L). Some *sfrp1a:NLS-Eos^+^* cells were also found in clusters 1 and 3, making up 13% and 29% of clusters 1 and 3, respectively. Clusters showed no differences in distribution among individual neuromasts across wild-type, *sost:NLS-Eos* and *sfrp1a:NLS-Eos* lines (Fig. S6D). Taken together, these results demonstrate that cells with distinct identities and localization can be classified by distinct cell shape characteristics.

### *atoh1a* mutants exhibit changes in cell shape across cell populations

We next sought to test what happens to cell shape distributions when a single cell type is absent. The transcription factor *atoh1a* is required for HC fate specification in zebrafish lateral line neuromasts (Milimaki et al., 2007; Itoh and Chitnis, 2001). We expected that the loss of the HC shape phenotype would be readily observable in *atoh1a* mutants. However, we wondered whether SCs would maintain the same shapes seen in wild-type fish or whether their shapes would also be altered in *atoh1a* mutants. In the *atoh1a:mRuby* line, the mRuby fluorophore was inserted into the coding sequence of the *atoh1a* gene, disrupting its function but acting as a readout for *atoh1a* promoter activity. This knock-in line enables comparisons not only between wild-type, mutant and heterozygous cell populations as a whole, but also between mRuby^+^ and mRuby^−^ populations in each genotype.

**Fig. 7.**
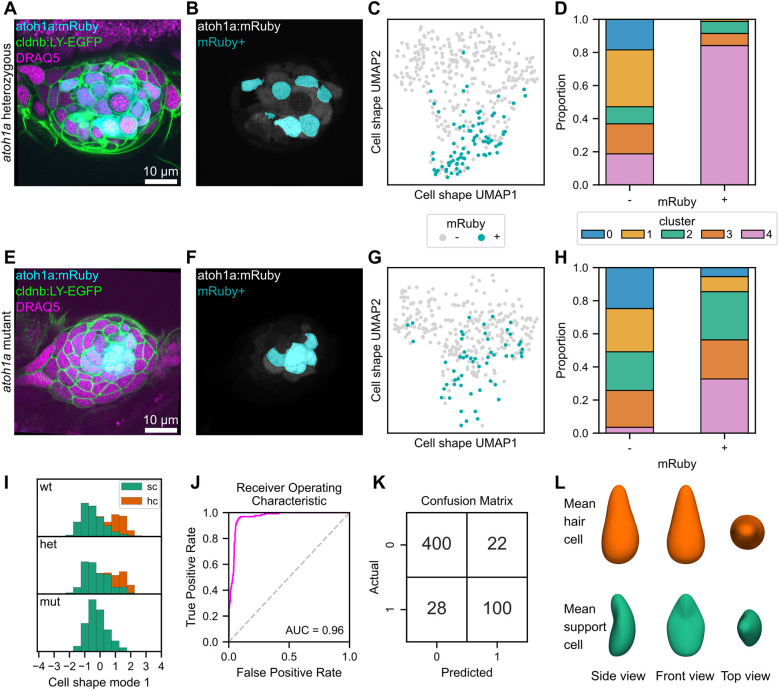
**Shape characteristics of *atoh1a:mRuby* heterozygotes and mutants, and logistic classifier for hair cells based on cell shape features.** (A-G) Analysis of cell shape distributions in *atoh1a:mRuby* heterozygotes (A-D) and mutants (E-H). (A,E) Single confocal slice of a neuromast from an *atoh1a:mRuby* heterozygote (A) and mutant (E) also expressing *Tg(-8.0cldnb:LY-EGFP)* and stained with DRAQ5. Image contrast was adjusted for visibility. (B,F) The same slices as in A and E, with the *atoh1a:mRuby* channel (gray) overlaid with a mask indicating cells classified as mRuby^+^ (cyan) for a *atoh1a:mRuby* heterozygote (B) and mutant (F). (C,G) UMAP of mRuby^+^ (cyan) and mRuby^−^ (gray) cells from *atoh1a:mRuby* heterozygotes (C) and mutants (F). (D,H) Proportions of mRuby^−^ cells (left bar) and mRuby^+^ cells (right bar) from *atoh1a:mRuby* heterozygotes (D) and mutants (H) in each cell shape cluster. (I) Distributions of cell shape mode 1 scores, separated by genotype (top row, wild type; middle row, *atoh1a* heterozygous; bottom row, *atoh1a* mutant). Hair cells (hc, green); support cells (sc, orange). (J) Receiver operating characteristic (ROC) curve (fuchsia) for a logistic regression classifier trained to detect hair cells based on cell shape mode 1-8. The area under the curve (AUC) score is shown (0.96). Gray dashed line, performance of a random classifier. (K) Confusion matrix for the cell shape-based hair cell classifier. 0, support cells; 1, hair cells. Top left, true negatives; top right, false positives; bottom left, false negatives; bottom right, true positives. (L) The idealized mean cell shape for hair cells (top, orange) and support cells (bottom, green).

In heterozygous fish, mRuby^+^ cells are localized to the center of the neuromast (Fig. 7A;  Fig. S6E). Cells classified as mRuby^+^ had fluorescence with z-score greater than 1 (Fig. 7B;  Fig. S6C). Overall, the proportions of cells in each cluster were similar to those of wild-type fish (Fig. S6D,G). However, mRuby^+^ cells were differentially proportioned across clusters and highly enriched in cluster 4 (Fig. 7C,D), which contains a high proportion of hair cells (Fig. S6H,I).

In homozygous mutant fish, mRuby^+^ cells were still found in the center of the neuromast, even though they did not differentiate into HCs (Fig. 7E,F; Fig. S6E). There was no statistically significant difference in the proportion of mRuby^+^ cells between heterozygous and homozygous animals (*X*^2^=1.8, d.f.=1, *P*=0.18; Fig. S6F). mRuby^+^ cells in homozygotes were differentially distributed across clusters (Fig. 7G,H; Fig. S6D,G). Relative to wild type and heterozygotes, mutant fish had far fewer cells in cluster 4 (Fig. 7H;  Fig. S6D,G). By contrast, the number of mRuby^+^ cells in clusters 2 and 3 were expanded in mutants. For mRuby^−^ cells, cluster 2 was expanded in mutants relative to heterozygotes (Fig. 7D,H). These results suggest that loss of *atoh1a* function is associated with a loss of the HC shape phenotype, an effect mainly seen in mRuby^+^ cells. The loss of HCs did not result in the appearance of a new cell shape cluster but rather assignment of cells into existing shape clusters, mainly cluster 2. These results demonstrate that cell shape analysis can be used to query changes in phenotype in contexts where development has been perturbed. Additionally, these results show that in neuromasts some aspects of cell shape are genetically determined, rather than being solely due to other factors (such as position).

### Shape modes can be used to classify HCs

We next examined the distribution of CSM1 scores from *atoh1a* heterozygous and mutant fish. We found that the distribution of CSM1 scores in heterozygotes resembled that of wild-type fish, with HCs associated with positive scores (Fig. 7I, top and middle). By contrast, the distribution of CSM1 scores in homozygous mutants was no longer bimodal (Fig. 7I, bottom), was skewed negatively and corresponded to the distribution of SCs in wild-type and heterozygous animals. CSM1 therefore captured the variation in shape between the two broadly defined neuromast cell populations (HCs and SCs) and was predictably perturbed in mutants lacking HCs.

The distinction between HCs and SCs in CSM1 scores suggested that cell shape modes may contain sufficient information for classification. We trained a logistic regression classifier to predict HC identity given the first eight cell shape mode scores (CSM1-8). The Receiver Operating Characteristic (ROC) curve indicated robust classifier performance with Area Under the Curve (AUC) of 0.96 (Fig. 7J). Despite the imbalance of classes in the dataset (77% SCs and 23% HCs), the classifier correctly labeled most (78%, 100/128) of the HCs in the test set, whereas few SCs (5%, 22/422) were incorrectly labeled as HCs (Fig. 7K). We also asked whether HCs could be classified using nucleus shape features, training a classifier using the first four nucleus shape modes (NSM1-4). The nuclear shape classifier performed similarly well to one using cell shape features (AUC score=0.0.96; data not shown). Therefore, nuclear shape features can also be used to classify HCs from SCs.

To visualize the shape characteristics associated with HC identity, we created a 3D reconstruction of the idealized mean HC and SC in the dataset by calculating the mean CSM1-8 scores for each cell type and deriving the corresponding SHE coefficients (Fig. 7L). As expected, the ‘average’ HC has a flask-like morphology, whereas the ‘average’ SC has a concave shape. These results demonstrate the utility of cell shape features both for describing and classifying cell types.

## DISCUSSION

Cells transition through states as they grow, divide and differentiate. Measuring gene transcription has proven to be a powerful method for understanding and classifying cell types. However, cells can change in ways that are not necessarily reflected in RNA transcript levels (Marklein et al., 2016; Gerbin et al., 2021). Cell shape analysis offers another avenue for quantitatively characterizing and classifying cells. Methods to encode cell shape in an unbiased high-dimensional representation have been employed with great success to cells in culture (Pincus and Theriot, 2007; Keren et al., 2008; Viana et al., 2023). However, most studies of cell shape in living developing animals have relied on 2D projections of cells or representative 3D geometric features, which may not capture many aspects of cell shape in these tissues. In this study, we use SH and PCA to encode cell shape information in a more complete, unbiased fashion, allowing data-driven analysis of the relationships between cell shape and fate.

A primary goal of our study was to understand the relationship between neuromast cell shape and established indicators of cell identity. If cell shape and markers for neuromast cell fate were tightly correlated, then it might be possible to classify cell types using shape features. Our results suggest that this relationship was true of HCs, as we were able to build a logistic classifier for HCs using our shape modes. When we performed unsupervised clustering of cells in shape space, there was a high degree of overlap between HC identity and one of the clusters (HCs comprised 79% of cluster 4). We believe similar methods could be used to build classifiers for cell types in other developmental contexts, potentially allowing cell fate to be predicted from image-based data without expression of cell fate markers.

The association between cell shape and SC subtypes was more nuanced. In our clustering analysis, DV cells defined by *sost:NLS-Eos* and peripheral cells defined by *sfrp1a:NLS-Eos* were enriched in distinct clusters. However, these clusters also contained substantial proportions of cells not expressing the marker. Given that SCs act as multipotent progenitors (Thomas and Raible, 2019; Romero-Carvajal et al., 2015; Viader-Llargués et al., 2018), we speculate that perhaps the more continuous distribution of SC shapes may reflect this plasticity. In contrast, HCs are a terminally differentiated cell type, which may be represented in their more distinct cell shape phenotype. A caveat is that our cutoff for considering a cell positive for a marker was relatively strict. In addition, our transgenic reporters may not always reflect the cells most recently expressing the transgene due to the high stability of the NLS-Eos protein (Cruz et al., 2015). Future studies using other methods to label SC subpopulations with genetic markers, such as RNA-FISH, could further elucidate the connection between gene expression and cell shape in neuromasts. More broadly, cell shape analysis could be integrated with other cellular properties, such as RNA transcript expression, protein expression and organelle location.

We were also interested to see whether nuclear shape could be used to classify cell types. Because nuclei are often easier to label and segment, nuclear shape representations may be more readily attainable in some developmental biology contexts. We found that nuclear shape could be used to reliably distinguish HCs from SCs. However, compared with cell shape features, nuclear shape features did not appear to distinguish as strongly between the SC subpopulations we tested in this study. However, nucleus shape may still be informative in other ways – we observed spatial patterns in nucleus shape within neuromasts, which may represent differences in cell type or state that were not tested in this study. Analysis of nucleus shape may also be more amenable to other tissues where cell membranes may not be easily resolved, allow more rapid collections of larger datasets, or allow analysis of cell types whose cell shapes are not amenable to SH analysis. More broadly, we imagine that cell shape, nuclear shape, or a combination of the two may be useful as predictors of cell fate in different tissues.

In many developmental contexts, it remains unclear how genetic factors determine cell shape. We observed that loss of function in *atoh1a* results in the large reduction of the cluster associated with HCs and concurrent increases in existing clusters, but does not lead to generation of a new shape cluster. Similarly, during an RNAi screen for morphological complexity in *Drosophila* Kc cells, RNAi knockdown typically did not generate new shapes but instead changed distributions of pre-existing wild-type shape states (Yin et al., 2013). It would be illuminating to explore other genes and signaling pathways that may pattern cell shape within neuromasts. For example, Fgf signaling has been shown to be required for apical constriction and rosette formation in the lateral line primordium (Harding and Nechiporuk, 2012; Ernst et al., 2012). Applying cell shape analysis to query other genetic and pharmacological perturbations could elucidate how cell shape in neuromasts is regulated by different signaling pathways.

Previous work has proposed that HC death might induce cell shape changes and tissue deformations that trigger SCs to proliferate; this idea originates from observations that (1) mechanical strain and cellular spreading induce proliferation in some contexts (Chen et al., 1997; Watt et al., 1988; Benham-Pyle et al., 2015) and (2) SC shape change during HC death is positively correlated with the HC regeneration capability of an organism (Burns et al., 2013; Burns and Corwin, 2014). Mechanical cues and cell density might also act as a brake on proliferation during regeneration, similar to what has been observed during development in the murine utricle (Gnedeva et al., 2017). Quantifying cell shape change during HC death or targeted ablation of neuromast cells could help to determine the role of cell shape in regulating proliferation and regeneration. Of particular interest is Yap, a transcriptional co-activator that regulates cell proliferation downstream of mechanical cues (Piccolo et al., 2014) that are known to play a role in regulating proliferation during development and regeneration of HCs in mammals (Rudolf et al., 2020; Gnedeva et al., 2020; Kastan et al., 2021). Coupling cell shape analysis with reporters for Yap activity could shed light on whether cell shape change acts upstream of Yap-induced cell proliferation. Cell shape analysis could also be combined with techniques to measure and perturb mechanical forces.

Previously, Hartmann and colleagues (2020) analyzed 3D cell morphology in the migrating posterior lateral line primordium using a recently described method (ISLA-CBE) based on point cloud morphometry. A strength of ISLA-CBE is that rotational variation can be minimized using a pairwise distance transformation. In addition, ISLA-CBE is not limited to only cells that can be accurately spherically parameterized. However, ISLA-CBE does not easily allow cell shapes to be reconstructed from any point in feature space, which is an advantage of our method. We anticipate that both methods will be useful in the future, as the ideal method for cell shape encoding will likely depend on the context and goals of the study. Although the ISLA-CBE method of encoding cell shape was different, we observed some commonalities with this study. Our observations suggested that HCs represent a discrete morphological state from SCs, whereas SCs appeared to vary more continuously in shape. Similarly, Hartmann and colleagues (2020) observed a continuous spectrum of shapes, without distinct clusters, in the lateral line primordium. Together, these results suggest a model whereby, initially, cells in the lateral line vary continuously in shape, but as HCs differentiate, they emerge as a discrete shape. How early these patterns develop and whether they change over time or remain static during neuromast deposition and maturity, could be addressed in future studies.

Although our study looked only at homeostatic, mature neuromasts at one time point, we believe the cell shape representation used here could be valuable for integrating information across multiple spatial and temporal scales. Using time lapse imaging and cell tracking, it would be possible to study dynamic cell shape changes during HC development, death and regeneration. We envision that cell shape states could be ordered along trajectories that represent developmental time, analogous to pseudotime analysis employed in single cell RNA sequencing studies. In addition, shape analysis can be applied to understand the morphologies of whole tissues and organs (Dalmasso et al., 2022). Combining pseudotime analysis with tissue-scale measurements can elucidate the relationship between individual cell shape change and tissue/organ morphogenesis, as has been demonstrated in a study of amphioxus notochord development (Andrews et al., 2021). Quantifying how these relationships evolve as the animal grows could shed light into how individual cell shape changes and movements shape organs, as well as how, conversely, the shape of organs constrains individual cell shape. Comparative cell shape studies could also be done between different sensory epithelia in the same organism or even between different species or ages, which could inform evolutionary relationships.

### Limitations of the study

Our machine learning-based method to segment cells may well be specific to the labeling conditions of our transgenic line and our live-imaging methods. In addition, the method was only semi-automated and required some manual curation and correction. We may see improvement if we retrain the model with this expanded corrected dataset as the ground truth. However additional adjustments would likely be needed for cells labeled with other reagents. As more sophisticated methods for automated segmentation become available, e.g. Cellpose (Pachitariu and Stringer, 2022), alternative pipelines may require less supervision. We also note that the resulting cell and nuclear shapes will likely be influenced by the specific characteristics of fluorescent labels and segmentation algorithms used, which will in turn influence subsequent SH analysis. We therefore constrained our analysis to the same nuclear and membrane label across different fish lines.

In the SH-based approach used here, all points on the surface of the cell must be able to be mapped onto a sphere to generate accurate reconstructions. However, some of our cells are sufficiently concave that this is not possible, because a line drawn from the center to the outside of the surface would intersect the surface more than once. As this problem likely extends to other cell types beyond neuromasts, there is a need for other 3D cell shape analysis methods that can better represent complex concave shapes. Development and application of alternative methods for spherical parameterization and SH expansion offer one potential solution to this problem. For example, Dalmasso and colleagues (2022) represented cell shapes as signed distances from concentric spheres, which enabled them to represent concave structures of mouse limbs. Another method using 4D hyperSH enables multiple disjointed objects to be parameterized together, and also enables representation of shapes with gaps or holes (Pasha Hosseinbor et al., 2015). There are also alternatives to SH for data-driven 3D shape representations, including deep-learning approaches such as autoencoders (De Vries et al., 2022 preprint). With the rate of exciting developments in the field, we expect that many methods will be available to study cell shape in a variety of contexts in the future.

## MATERIALS AND METHODS

### Fish lines

Experiments were conducted on 5 dpf larval zebrafish (*Danio rerio*). The following zebrafish lines were used for these studies: *Tg(-8.0cldnb:LY-EGFP)^zf106Tg^* (RRID:ZDB-ALT-060919-2) (Haas and Gilmour, 2006), *sost^w215Tg^* (RRID: ZDB-ALT-190909-10) and *sfrp1a^w217Tg^* (RRID:ZDB-ALT190909-6) (Thomas and Raible, 2019), and *atoh1a^w271Tg^* (this paper). Animals were used at a stage before sex is determined.

### Generation of *atoh1a^w271Tg^*

The *atoh1a^w271Tg^* line was generated by CRISPR-Cas9 knock-in of the mRuby fluorophore into the coding sequence of the endogenous *atoh1a* locus, as previously published (Thomas and Raible, 2019; Kimura et al., 2014). Homozygotes exhibit phenotypes consistent with *atoh1a* loss of function, such as hearing and vestibular defects, and are viable until ∼10 days post fertilization (dpf).

### Fish handling

Before experiments, embryos and larvae were raised in E3 embryo medium [14.97 mM NaCl, 500 μM KCl, 42 μM NaHPO_4_, 150 μM KH_2_PO_4_, 1 mM CaCl_2_ dehydrate, 1 mM MgSO_4_ and 0.714 mM NaHCO_3_ (pH 7.2)] at 28.5°C. Zebrafish larvae were fed rotifers daily from 4 dpf onwards. Experiments used zebrafish at 5 dpf, a stage before sex is determined. Zebrafish experiments and husbandry followed standard protocols in accordance with the University of Washington Institutional Animal Care and Use Committee guidelines.

### DRAQ5 staining

On the day of imaging, DRAQ5 dye (Thermo-Fisher) was diluted in E3 embryo medium to a working concentration of 5 µM. 5 dpf larval zebrafish were incubated in this solution for ∼1 h, then prepared as described in the ‘Preparation of larvae for live imaging’ section. We observed the best results when the fish were incubated in DRAQ5 for no longer than 1 h and immediately prepared for imaging afterwards.

### Photoconversion of NLS-Eos

For experiments carried out with *sost:NLS-Eos* or *sfrp1a:NLS-Eos* lines, larvae were photoconverted using an iLumen 8 UV flashlight (purchased from Amazon) for 15-20 min. Photoconversion was carried out by placing fish in a 60×15 mm petri dish, removing the petri dish lid and placing the UV flashlight directly over the dish within a box lined with aluminum foil.

### Preparation of larvae for live imaging

Fish were transferred to E3 embryo medium containing ∼1.5 mM MESAB to be anesthetized. Once larvae were suitably anesthetized, they were mounted on slides with bridged coverslips in 1.2% low melting point agarose. Fish were mounted in a standard orientation of anterior facing left and dorsal upwards.

### Confocal imaging

Imaging was carried out using a Zeiss LSM 880 with Airyscan (Huff, 2015) equipped with a Zeiss C-Apochromat 40×/1.2 W numerical water objective. Imaging was performed at room temperature (∼25°C). For each fish, several anterior neuromasts were imaged. Neuromasts were chosen from a list based on which had the most ideal orientations in the fish being imaged (i.e. those neuromasts closest to the coverslip with apical-basal axes roughly parallel to the axial plane/*z*-axis of imaging). Lasers of 633 nm, 561 nm and 488 nm were used for excitation. Gain was adjusted for different transgenes but kept constant across experiments with the same transgene. Images were collected with physical voxel size of 0.05×0.05×0.22 µm^3^. All images were captured using the Zen Black acquisition software (Zeiss). Minimal cell death was observed during imaging; any neuromasts exhibiting significant amounts of cell death were excluded from further analysis.

### Image preprocessing

Using Zen Blue, Airyscan Processing was applied to all raw images using the default settings for pixel reassignment and deconvolution. Next, a custom Python script using the aicsimageio package (Brown et al., 2021) was used to remove any unneeded channels and export the CZI files to TIFFs. The original CZI files were kept to ensure access to the original acquisition metadata. To correct for *xy* drift between *z* slices, StackReg was used, either through the MultiStackReg FIJI plug-in or a custom script using the pystackreg Python port of StackReg (Thevenaz et al., 1998). Using the ‘rigid body’ method and ‘previous’ setting for the reference, transformation matrices were calculated for the membrane channel, saved and applied to all channels in the image. Images were manually inspected after stack registration to ensure appropriate drift correction.

Occasionally, imaging acquisition errors or *z* drift caused either duplication of *z* slices within the stack or the need to acquire additional *z* slices to cover the entire neuromast. In cases where this could be easily manually corrected, duplicated *z* slices were deleted, or two *z* stacks were combined to cover the entire neuromast. These manual corrections were carried out using FIJI (Schindelin et al., 2012) before stack registration.

### Training of machine learning models for detection of nuclei and membranes

Models to detect neuromast nuclei and membranes were trained using the iterative deep-learning workflow of the Allen Cell and Structure Segmenter (Chen et al., 2018 preprint). For the membrane model, preliminary segmentations of cell boundaries were initially generated using the lamin B1 workflow from the Segmenter. The ‘Curator’ tool from the Segmenter was used to mask out areas of the images that were not segmented accurately. The original membrane model was trained on a dataset of 14 neuromasts labeled with *Tg(-8.0cldnb:LY-EGFP)*. The membrane model predictions were thresholded and used as input for an interactive distance transform watershed segmentation workflow. Upon generating the label images, the find_boundaries function from scikit-image (van der Walt et al., 2014) was used to generate watershed lines, which was used as input to train a second membrane model with improved performance. This process was repeated once more with a new dataset of nine *Tg(-8.0cldnb:LYEGFP)* neuromasts stained with DRAQ5, except labeled nuclei were used to generate seeds for the watershed instead of the distance transform. The results were used to train the third membrane model, which was used for instance segmentation (delineation of individual objects) of all the images in this study.

To generate the nucleus mask model, the ‘H2B coarse’ model provided by AICS^22^ was used to generate preliminary nucleus predictions. The predictions were thresholded and the membrane intensity image (or membrane boundary predictions) were used to ‘split’ the nuclei (as described in ‘Instance segmentation’). Similar to the refinement for the membrane model, a distance transform watershed workflow was applied to thresholded nuclei predictions to generate labeled nuclei, which were converted to binary masks and used to train the first nucleus mask model. This process was repeated (starting from the model 1 predictions instead of the H2B coarse model predictions) to generate a second nucleus mask model with improved performance, which was used for all the images in this study.

### Instance segmentation

Instance segmentation of nuclei was carried out in a semi-automated interactive fashion using a custom Python script. The Python packages napari (Sofroniew et al., 2022) and magicgui (Lambert et al., 2023) were used to create a graphical user interface for this step, enabling parameter tuning, interactivity and manual annotations when needed.

First, the nucleus mask predictions and cell boundary predictions were binarized using thresholds of 0.6 and 0.4, respectively. These thresholds were selected as a balance between capturing intensity signal corresponding to the structures of interest and minimizing artifacts due to noise. The binarized membrane predictions were used to ‘split’ falsely merged nuclei in the binarized nucleus mask predictions, using one of two methods. For experiments not involving NLS-Eos transgenes, the nucleus mask splitting was carried out fully automatically (e.g. wherever the membrane mask value was 1, the corresponding pixels in the nucleus mask were set to 0). For experiments involving NLS-Eos transgenes, incomplete photoconversion and/or ongoing transgene expression can cause some signal in the nuclei in the green channel, where the membranes are also imaged. This overlap can create artifacts when the cell boundary model is applied. To circumvent the issue, an alternative ‘interactive’ nucleus splitting strategy was devised. For interactive nucleus splitting, regions of interest (ROIs) were manually drawn slice by slice in areas where splitting was desired using the shapes tool in napari. Only these regions within the membrane mask were then considered during the splitting process.

Following nucleus splitting, individual nuclei were labeled using distance transform watershed. First, the Euclidean distance transform of the nucleus mask was calculated and smoothed using a Gaussian kernel. Next, the local maxima of the smoothed distance transform were calculated using the peak_local_max function from scikit-image. The local maxima were then used as markers for a watershed segmentation using the inverted distance transform as the input. The sigma parameter for the Gaussian smoothing of the distance transform and the minimum distance parameter for the peak_local_max function were adjusted interactively to yield the best results for each image. Remaining segmentation errors were corrected by manual annotation using a Wacom Cintiq 16 drawing tablet and built-in labels layer tools in napari.

To segment cells, the segmented nuclei were used as markers for a watershed segmentation (Beucher and Lantuéjoul, 1979) with the cell boundary predictions (i.e. the raw unthresholded output of the membrane model inference) as input. The resulting preliminary cell labels were post-processed to remove small objects and manually corrected using a Wacom Cintiq 16 drawing tablet and built-in labels layer tools in napari. After segmentation, each field of view was manually inspected and some cells were excluded for quality control (e.g. due to having poor signal or extending beyond the edge of the field of view). One-hundred and sixty-four cells were manually excluded in this way.

### Cell dataset preparation

Before running the cell variance analysis pipeline (Viana et al., 2023), individual cells from the raw and labeled whole neuromast images were cropped, subsampled in z to obtain isotropic voxel size, and saved as individual images. A custom Python script was used to prepare cropped single cells and a manifest.csv file compatible with the pipeline.

### Cell and nucleus alignment

Cells were aligned using a series of three rotations. The first rotation was carried out with reference to the neuromast organ. The neuromast centroid was calculated from a mask representing the whole neuromast (created from the cell segmentation channel). For each cell, the relative position of the cell centroid to the neuromast centroid was used to calculate the angle between the cell centroid and the *x*-axis. The corresponding cropped image of the cell was then rotated around the *z*-axis by this angle to minimize variation originating from the rosette structure of the neuromast.

The second and third rotations were carried out with reference to the cropped cell only. For the second rotation, the cropped image of the cell was rotated such that the long axis of the cell projected in the *xz*-plane was aligned to the *z*-axis. For the second rotation, the cropped image of the cell was rotated such that the long axis of the cell projected in the *yz*-plane was aligned to the *z*-axis, unless this angle was greater than 45°. In that case, the *yz* long axis was aligned to the *y*-axis instead. This adjustment prevented cells from being incorrectly rotated by 90° if their long axis was in the horizontal plane.

To generate the nucleus shape space, the nuclei were aligned separately from the cells. The procedure was the same as for the cells, except for the second rotation, which also was constrained to be no greater than 45°. If the angle were greater than 45°, the long axis of the nuclei in the *xz*-plane was aligned to the *x*-axis instead of the *z*-axis.

### Spherical harmonics parameterization

After cell dataset preparation and alignment, SH coefficients for each cell and nucleus were calculated using the cvapipe_analysis package (Viana et al., 2023) First, the ‘loaddata’ step was run with the prepared manifest.csv, then the ‘computefeatures’ step was run. To determine the degree of expansion (L_max_) for SH parameterization, we plotted several metrics of reconstruction fidelity for different values of L_max_ using a subset of the data. As L_max_ increases, shapes can be reconstructed with greater detail, but higher L_max_ values incur additional computational costs and exhibit diminishing returns in reconstruction fidelity. We selected an L_max_ of 32 as a value around which reconstruction fidelity plateaued, which yielded 2178 coefficients. The directed Hausdorff distance (Huttenlocher et al., 1993), which represents the furthest distance between the closest points on the original and reconstructed meshes, was used as a measure of reconstruction error. A Gaussian mixture model was used to identify cells belonging to the second peak in the reconstruction error distribution, which were subsequently excluded from cell shape analysis.

### Principal components analysis and unsupervised clustering

Principal components analysis (PCA) was performed using the Python library scikit-learn (Pedregosa et al., 2011). To create ‘shape modes’, PCs were *z*-scored (i.e. the values for each PC were divided by their respective standard deviation). Visualizations of cells in shape space were generated by applying the inverse PCA transform to generate corresponding SHE coefficients, which were then used to generate 3D reconstructions using the aics-shparam package. Reconstructions were visualized in 3D using ParaView (Ahrens et al., 2005).

Clustering was performed using the Python package Phenograph (Levine et al., 2015) with the Leiden algorithm for community detection (Traag et al., 2019). For analysis of cell shape, the first eight PCs were used for clustering; for nuclear shape, the first four PCs were used. These PCs explained 70% and 76% of total variance, respectively. The Uniform Manifold Approximation and Projection (UMAP) implementation from the Python package umap-learn was used to project the data in two dimensions for visualization (McInnes et al., 2018). Partition-based graph abstraction (PAGA) was performed using the scanpy Python package (Wolf et al., 2018, 2019). PAGA was used to initialize the layout of UMAPs.

### Quantification of fluorescence intensity of transgenes

For reporter lines expressing nuclear-localized Eos, *sost:NLS-Eos* and *sfrp1a:NLS-Eos*, the mean intensity within each cell was calculated using the segmented nuclei as masks. For *atoh1a:mRuby*, which has cytoplasmic mRuby localization, the mean intensity within each cell was calculated using the segmented cell as masks. Because intensities of fluorescent proteins from targeted insertions can vary between individual neuromasts and fish, we z-scored the mean intensities for each neuromast and used a threshold of 1 to determine cells ‘positive’ for a marker. In other words, the mean intensity of fluorescent protein within a cell needed to be at least one standard deviation above the mean for that neuromast to be classified as ‘positive’ for that protein.

### Measurement of cell location and shape features

Locations of neuromast cells were estimated using the *x* and *y* coordinates of the cell segmentation centroids. The neuromast centroid was calculated by using the cell segmentation channel to generate a mask representing the whole neuromast. For each cell, the distance between the cell centroid and neuromast centroid in the *xy* plane was calculated. This distance was normalized to that of the cell with the greatest distance from the neuromast centroid to account for differences in neuromast size (Vlader-Llargués et al., 2018).

We used the first alignment rotation angle to approximate the angular location of each neuromast cell. This angle represents the angle between the neuromast *x*-axis (which extends from the neuromast centroid) and a vector drawn from the cell centroid to the neuromast centroid in the *xy*-plane (Fig. 2B). In our images, the neuromast *x*-axis is approximately parallel to the anterior-posterior body axis of the fish. Lateral line neuromasts can be polarized in one of two ways relative to the anteroposterior body axis: parallel or perpendicular (López-Schier et al., 2004). Because our dataset contained neuromasts of both types, we standardized the orientations of the neuromasts by adjusting the angles of perpendicular neuromasts using the following formulae:

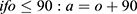


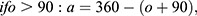
where *o* is the original angle and *a* is the adjusted angle. This is equivalent to rotating a perpendicular neuromast 90° counterclockwise such that its dorsoventral axis is aligned with the anteroposterior body axis. The decision to rotate counterclockwise was based on the fact that Emx2 is expressed in the anterior region of parallel neuromasts and the dorsal region of perpendicular neuromasts (Jiang et al., 2017). The radial distances and angular locations of each cell were combined to plot the locations of neuromast cells on polar coordinates. Other shape measurements, such as volume, height, etc., were obtained as outputs of the cvapipe_analysis computefeatures step (Viana et al., 2023)

### Logistic regression classification

Logistic regression was performed using the Python package scikit-learn (Pedregosa et al., 2011). The data were split into an 80/20 ratio of training data to test data. The test and train subsets were split such that both had similar proportions of HCs. The logistic regression model was trained to classify HCs on the train subset, using one or more shape modes as predictor variables. The trained model was then applied to the testing subset to generate the ROC curves and confusion matrices.

### Statistical analysis

χ^2^ analysis was used to distinguish distribution differences among groups, using the Python package SciPy (Virtanen et al., 2020).

## Supplementary Material



10.1242/develop.202251_sup1 Supplementary information
