# Supplementary material for: Spherical harmonics analysis reveals cell shape-fate relationships in zebrafish lateral line neuromasts
Source: Development. 2024 Jan 26;151(2):dev202251. doi: 10.1242/dev.202251 (PMC10905750; doi:10.1242/dev.202251)
Supplement: Supplementary information [file develop-151-202251-s1.pdf]

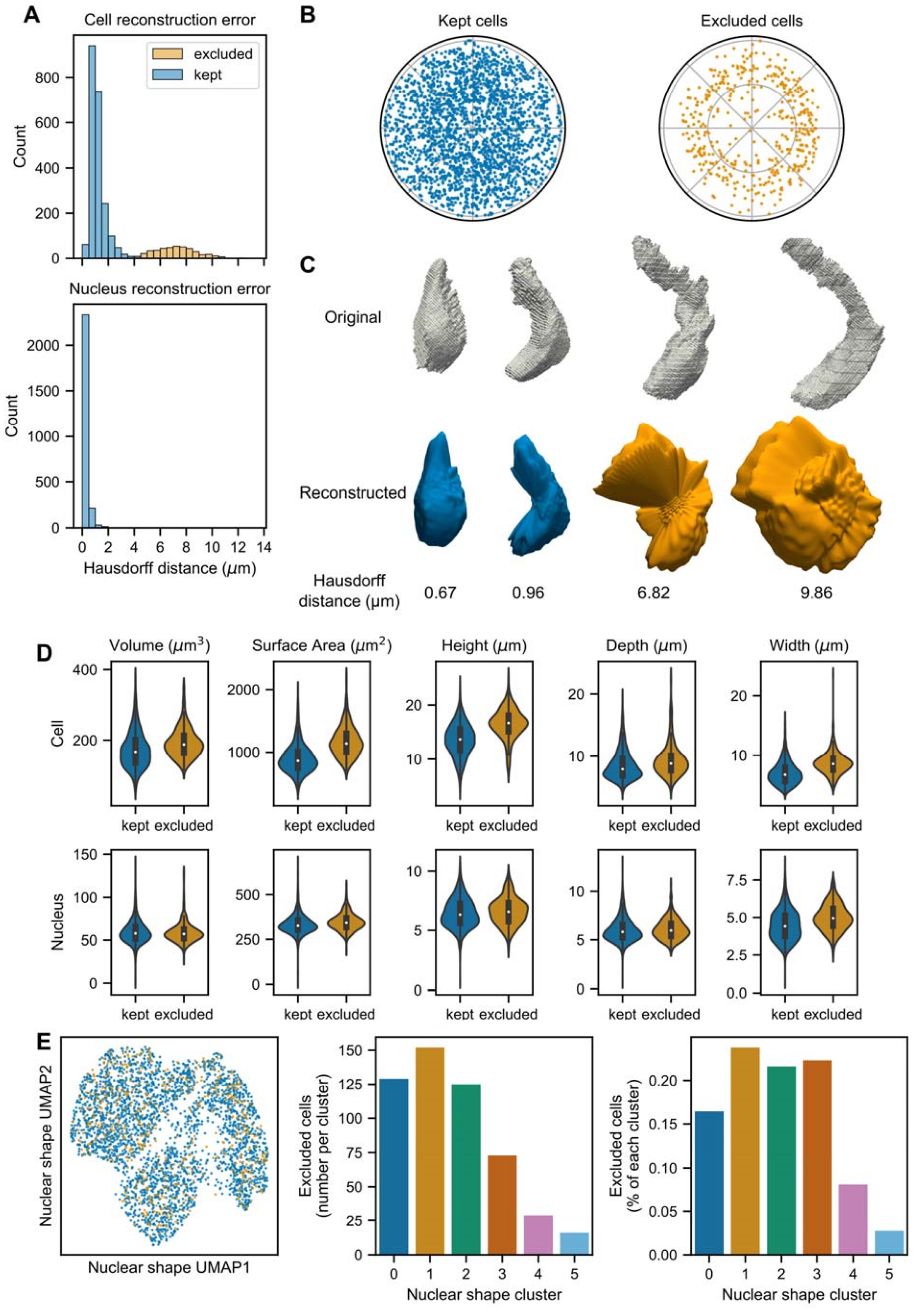

Development • Supplementary information

Fig. S1.

(A) Distribution of reconstruction error, as measured by directed Hausdorff distance, for cell shape (top) and nuclear shape (bottom). Some cells were excluded from cell shape analysis (orange) due to high reconstruction error.

(B) Polar plots showing relative locations of cells kept (left, blue) and excluded from cell shape analysis (right, orange).

(C) Examples of well reconstructed (left) and poorly reconstructed (right) cells. 3D representations of the original mesh (top), reconstructed mesh (middle), and the associated Hausdorff distances (bottom) for two examples of each are shown.

(D) Distributions of cell (top) and nuclei (bottom) shape features in kept (blue) and excluded (orange) cells.

(E) Left: UMAP projection of cells in nuclear shape space (corresponding with Figure 5A) colored by whether they were kept (blue) or excluded (orange) from cell shape analysis. Middle: Excluded cells plotted as number

(raw counts) for each nuclear shape cluster. Right: Excluded cells as a percentage of each nuclear shape cluster.

Development • Supplementary information

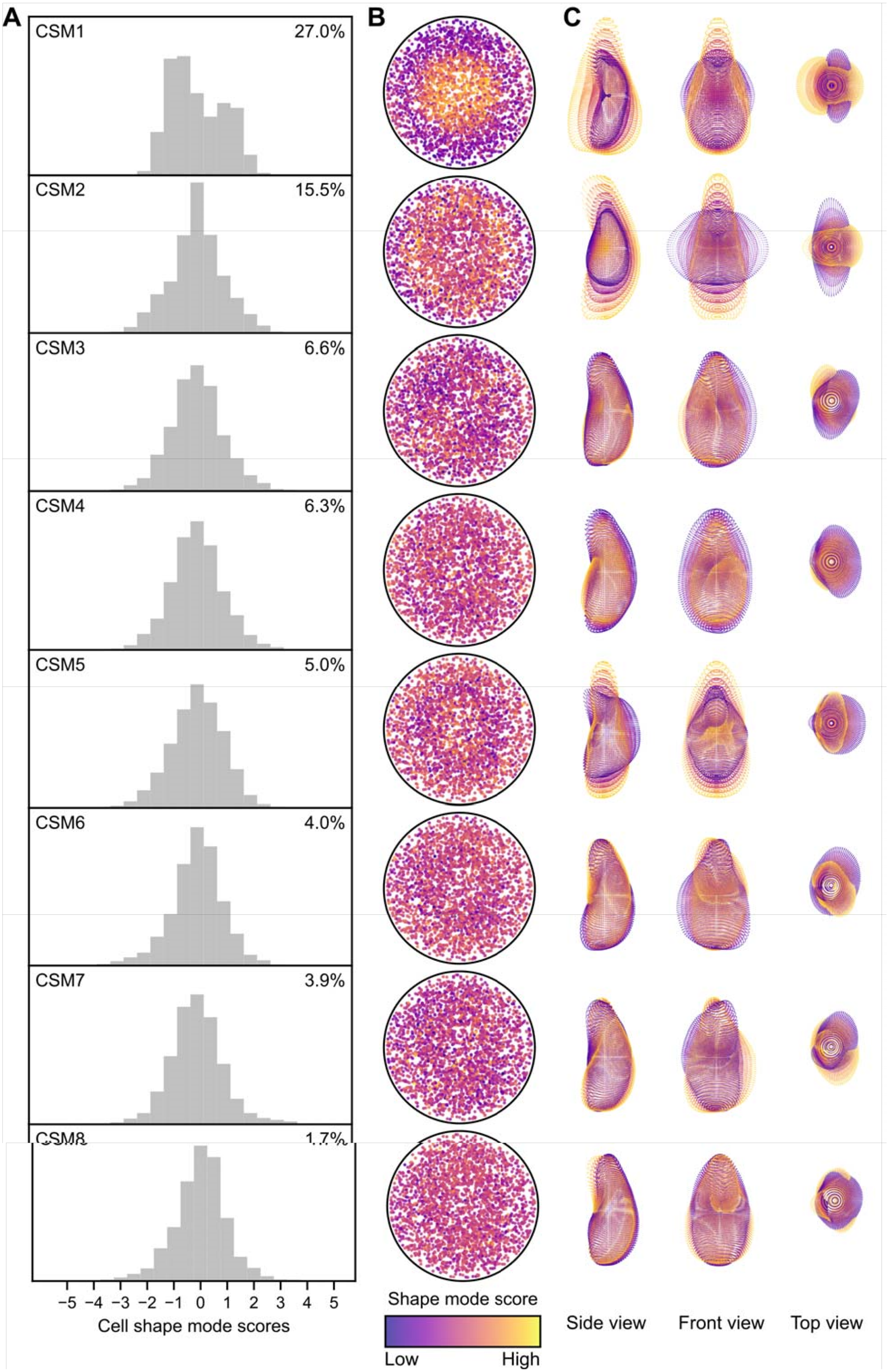

Development • Supplementary information

Fig. S2.

(A) Distributions of shape mode scores for the first eight cell shape modes (CSM1-CSM8). Percent variance explained for each shape mode is provided in the upper right corner.

(B) Polar plots showing relative locations of neuromast cells in the dataset, color coded by shape mode scores for CSM1-CSM8.

(C) Overlaid 3D point view representations of CSM1-CSM8, generated by finding the mean cell and varying each shape mode by up to 2 standard deviations (sd) in increments of 1 sd while holding other shape modes at the mean (0).

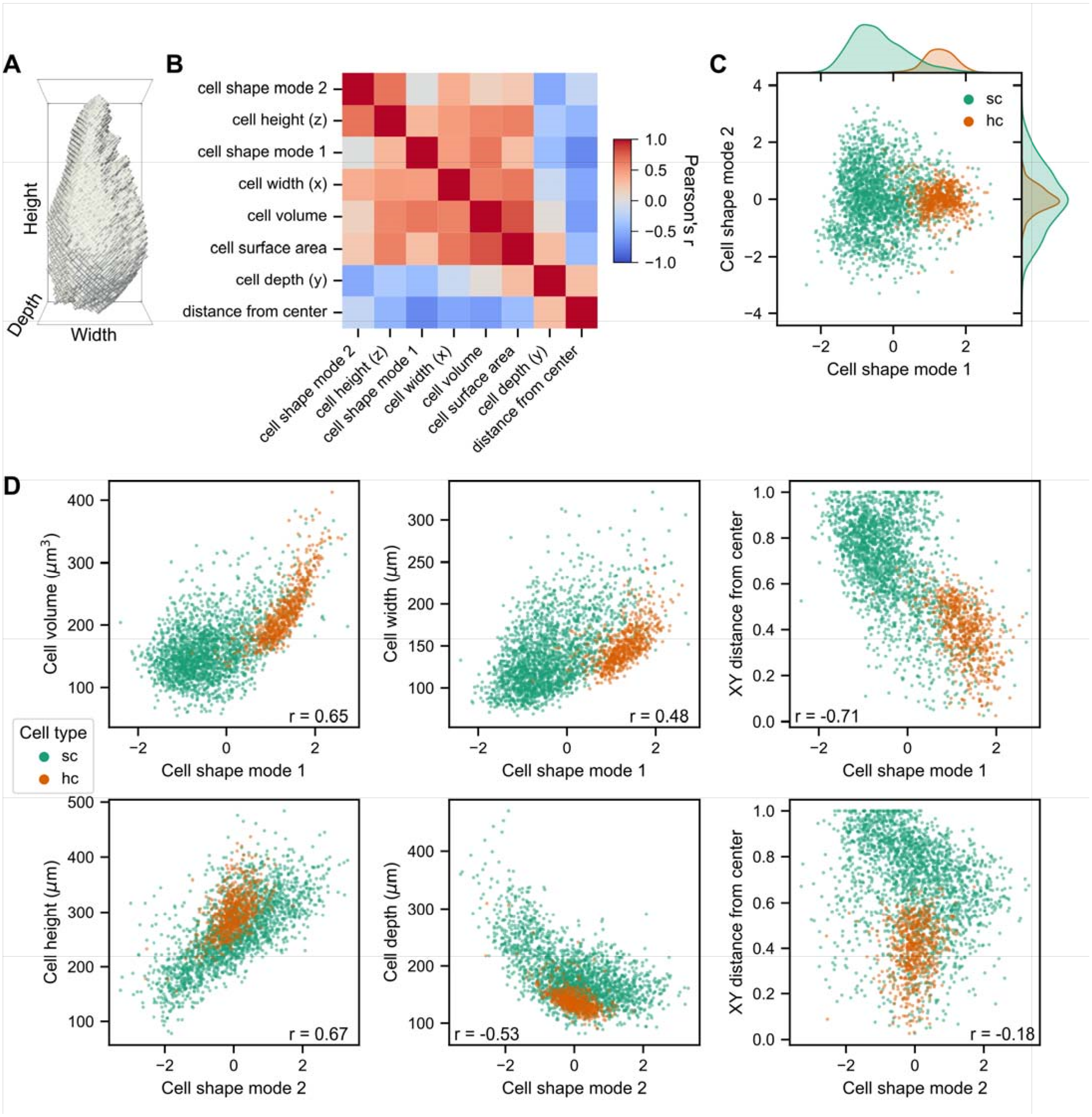
A
Height
Depth
Width
B
cell shape mode 2
cell height (z)
cell shape mode 1
cell width (x)
cell volume
cell surface area
cell depth (y)
distance from center
Pearson's r
C
Cell shape mode 1
Cell shape mode 2
sc
hc
D
Cell volume (μm³)
Cell width (μm)
XY distance from center
Cell height (μm)
Cell depth (μm)
r = 0.65
r = 0.48
r = -0.71
r = 0.67
r = -0.53
r = -0.18
Cell type
sc
hc

Fig. S3.

(A) Side view of a 3D projection of a representative cell with its dimensions labeled.

(B) Heatmap of correlations between cell shape mode 1, cell shape mode 2, and cell shape/location features.

(C) Plot of cell shape mode 1 and cell shape mode 2 for SCs (green) and hair cells (orange).

(D) Top row: Correlations between cell volume (left), width (center), and distance from center (right) versus cell shape mode 1 color coded by cell type. Bottom row: Correlations between cell height (left), depth (center), and distance from center (right) versus cell shape mode 2 color coded by cell type. The Pearson correlation coefficient (r) is displayed on each plot. SCs (green) and hair cells (orange).

Development • Supplementary information

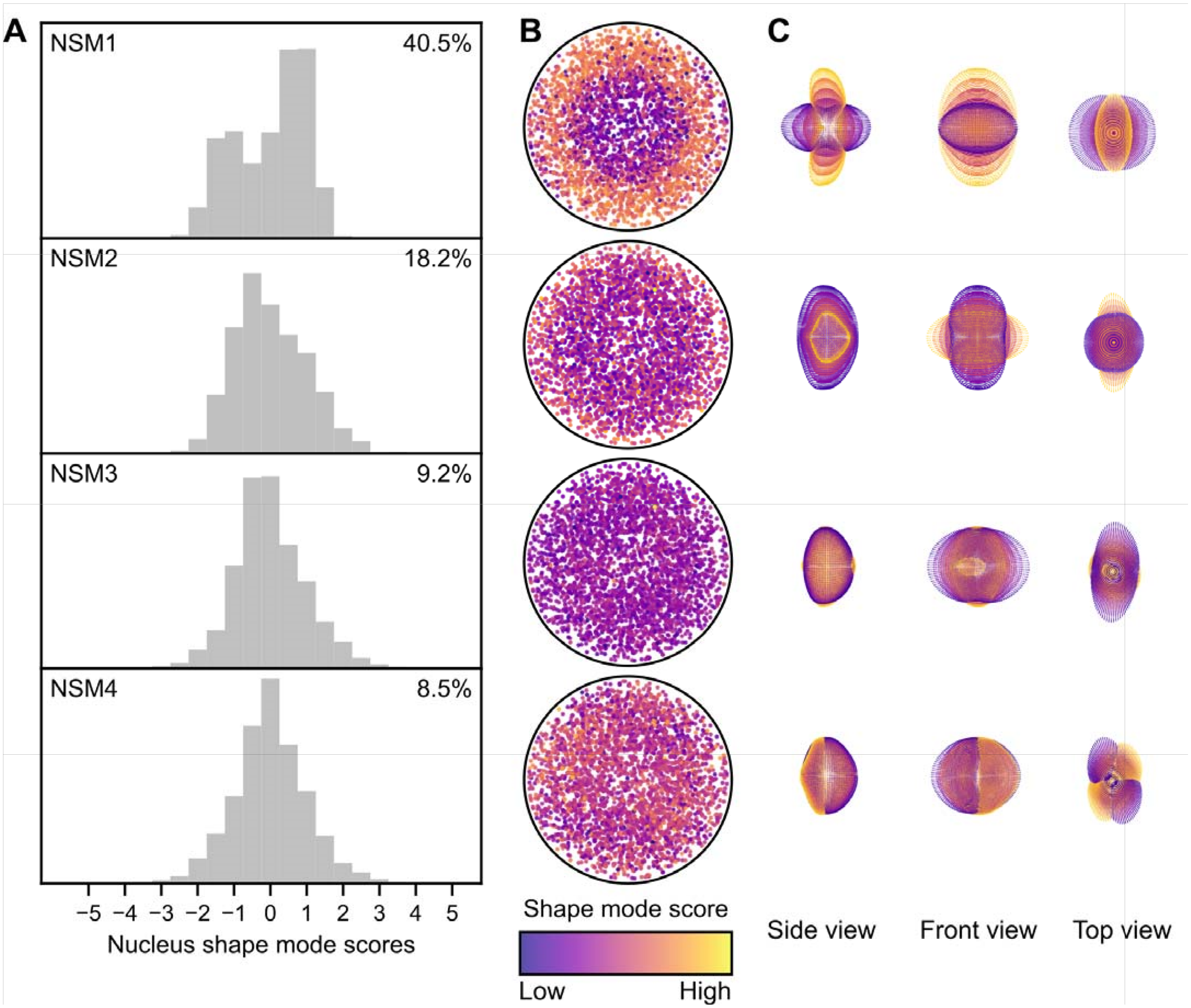

Fig. S4.

(A) Distributions of shape mode scores for the first four nucleus shape modes (NSM1-NSM4). Percent variance explained for each shape mode is provided in the upper right corner.

(B) Polar plots showing relative locations of neuromast cells in the dataset, color coded by shape mode scores for NSM1-NSM4.

(C) Overlaid 3D point view representations of CSM1-CSM8, generated by finding the mean cell and varying each shape mode by up to 2 standard deviations (sd) in increments of 1 sd while holding other shape modes at the mean (0).

Development • Supplementary information

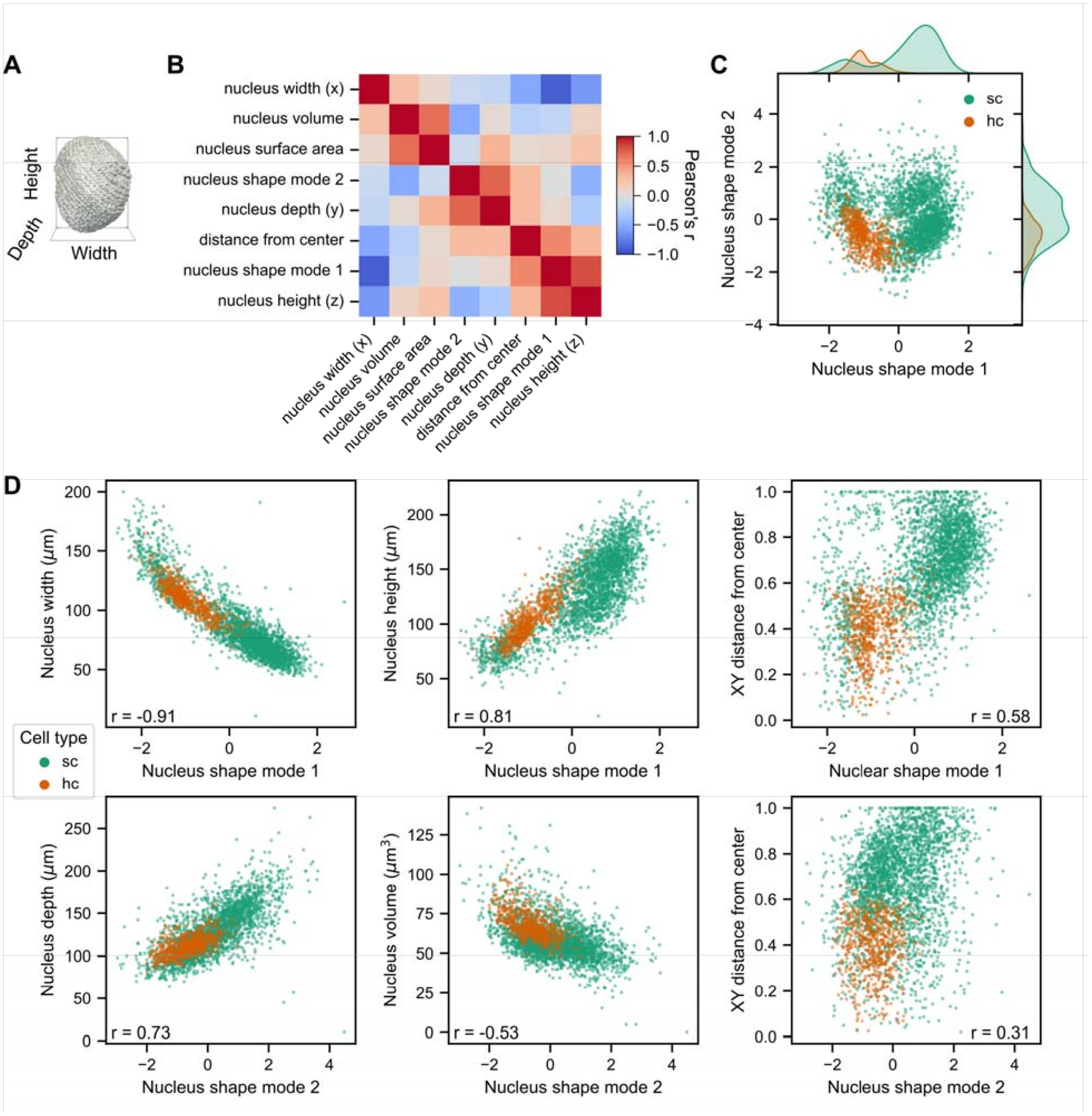

Fig. S5.

(A) Side view of a 3D projection of a representative nucleus with its dimensions labeled.

(B) Heatmap of correlations between nucleus shape mode 1, nucleus shape mode 2, and nucleus shape/location features.

(C) Plot of nucleus shape mode 1 and nucleus shape mode 2 for SCs (green) and hair cells (orange).

(D) Top row: Correlations between nucleus width (left), height (center), and distance from center (right) versus nucleus shape mode 1 color coded by cell type. Bottom row: Correlations between nucleus depth (left), volume (center), and distance from center (right) versus nucleus shape mode 2 color coded by cell type. The Pearson correlation coefficient (r) is displayed on each plot. SCs (green) and hair cells (orange).

Development • Supplementary information

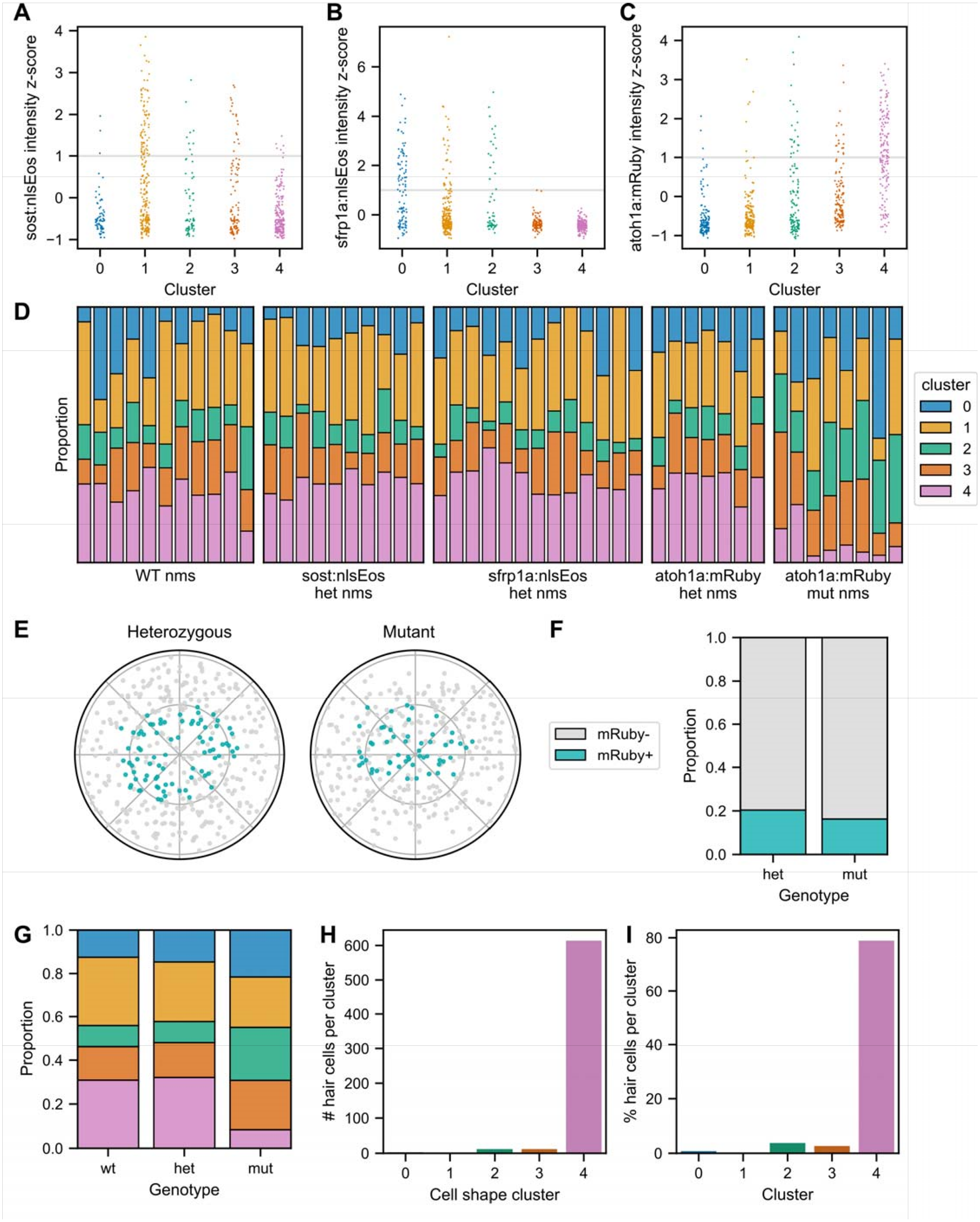

Development • Supplementary information

Fig. S6.

(A) Distribution of mean *sost:NLS-Eos* intensities (z-scored) for cells in each cell shape cluster. Gray line represents intensity threshold (1 SD) used to label a cell as positive.

(B) Distribution of mean *sfrp1a:NLS-Eos* intensities (z-scored) for cells in each cell shape cluster. Gray line represents intensity threshold (1 SD) used to label a cell as positive.

(C) Distribution of mean *atoh1a:mRuby* intensities (z-scored) for cells in each cell shape cluster. Gray line represents intensity threshold (1 SD) used to label a cell as positive.

(D) Proportions of cells in individual neuromasts belonging to each cluster, grouped by reporter expression and genotype (n=49 neuromasts).

(E) Polar plots of relative cell locations of mRuby- cells (gray) and mRuby+ cells (dark cyan) in *atoh1a:mRuby* heterozygotes (left) and *atoh1a:mRuby* mutants (right).

(F) Proportions of mRuby+ cells (dark cyan) and mRuby- cells (gray) in *atoh1a:mRuby* heterozygotes (left bar) and *atoh1a:mRuby* mutants (right bar).

(G) Proportions of cells of each genotype - *atoh1a:mRuby* mutants, heterozygotes, and wildtype - belonging to each cell shape cluster.

(H) Hair cells as raw counts (number) within each cell shape cluster.

(I) Hair cells as a percentage of each cell shape cluster.

Development • Supplementary information

Table S1. Summary of the dataset. Each batch represents a separate imaging session. All neuromasts were labeled with DRAQ5 dye in the *Tg(-8.0cldnb:LY-EGFP)* background; the “reporter” column indicates additional genetic labels. “Filter” refers to cells excluded for having high cell shape reconstruction error (see Fig. S1 for details).

batch	reporter	# neuromasts	# cells (pre-filter)	# cells (post-filter)
1	sfrp1a:nlsEos	7	453	383
2	sost:nlsEos	10	824	676
3	sfrp1a:nlsEos	6	459	371
4	atoh1a:mRuby	10	560	496
5	atoh1a:mRuby	5	295	244
6	N/A (wildtype)	11	683	580
