# Supplementary material for: Spherical harmonics analysis reveals cell shape-fate relationships in zebrafish lateral line neuromasts
Source: Development. 2024 Jan 26;151(2):dev202251. doi: 10.1242/dev.202251 (PMC10905750; doi:10.1242/dev.202251)
Supplement: Supplementary file 1 [file develop-151-202251_review_history.pdf]

Spherical harmonics analysis reveals cell shape-fate relationships in zebrafish lateral line neuromasts

Madeleine N Hewitt, Iván A Cruz and David W Raible
DOI: 10.1242/dev.202251

Editor: Steve Wilson

Review timeline
Original submission: 11 August 2023
Editorial decision: 11 September 2023
First revision received: 4 December 2023
Accepted: 28 December 2023

Original submission

First decision letter

MS ID#: DEVELOP/2023/202251

MS TITLE: Data-Driven 3D Shape Analysis Reveals Cell Shape-Fate Relationships in Zebrafish Lateral Line Neuromast

AUTHORS: Madeleine N Hewitt, Ivan Cruz, and David W Raible

I have now received two referees reports on the above manuscript, and have reached a decision. The referees' comments are appended below, or you can access them online: please go to BenchPressand click on the 'Manuscripts with Decisions' queue in the Author Area.

The overall evaluation is positive and the refereesdon't have many suggestions for changes. However, they don't think that the study yields much in the way of novel biological insights and so its greater value to the community will be the detailed description of the approach you use for the in vivo analyses. Consequently, I think the study would be more appropriate as a Techniques and Resources paper than a research article and wewould be happy to publish a revised manuscript in Development in this format.

Please attend to all of the reviewers' comments in your revised manuscript and detail them in your point-by-point response. If you do not agree with any of their criticisms or suggestions explain clearly why this is so.

Reviewer 1

Advance summary and potential significance to field

This manuscript presents a technical tour-de-force aimed at quantitatively classifying the shape of every cell in an organ in its natural context. On this front, the work is ambitious and necessary. The quality of the data is excellent and the presentation is also very good. Therefore, the results raise the bar in the field and serve as a recipe to follow. The biological insight, however, is extremely limited, mainly because most of (if not all) the conclusions are either a refresher of known facts or small details that were previously unknown. For instance, although it is true that "The distinction between hair cells and support cells was discrete and accounted for much of the variation in

© 2024. Published by The Company of Biologists under the terms of the Creative Commons Attribution License (https://creativecommons.org/licenses/by/4.0/).

neuromast cell and nucleus shape..." had not been quantified before, anyone with experience looking at neuromasts can easily tell SCs and HCs apart qualitatively.

Comments for the author

One things that I am really curious about is why the authors did not trace the same cell over time to test whether a specific cell can indeed be recognised as such at any particular moment?

Also, why the authors did not generate a clone to mark some isolated cells (say in red) and segment them, the same cells but separately, using the green and the red membrane markers. This is to understand better how limiting or confounding the assessment of cell shape may be when looking at a single marker.

pp3. second paragraphs, it is unclear what "the method", stated twice, refers to.
SH or PCA?
Third paragraph, why do authors call SCs "glia like"? I saw this being done before (Zhang et al., 2018), but I think that it is a mistake because it will confuse the non-expert.

The authors rely exclusively on the Tg(cldnb:LY-EGFP) transgenic line for cell-shape acquisition. I wonder whether possible heterogeneity in the expression level of this transgene across the epithelium may impact segmentation and by extension cell-shape classification.

pp4. second paragraph "...good quality segmentations for most cells". How was quality assessed to call it good?

pp5. I do not understand how CSM2 differentiates among SCs, which are less variant that SCs from HCs.

pp8. The authros state that "Taken together, these results demonstrate that cells with distinct identities, as indicated by sost:NLS-Eos and sfrp1a:NLS-Eos expression, can be classified by distinct cell shape characteristics."

However, this may be indirect and a simple consequence of localization in the neuromast rather than identity.

<u>Reviewer 2</u>

Advance summary and potential significance to field

The study by Hewitt et al provides a template for analysis of 3D shape data collected from high resolution confocal imaging of organogenesis in live embryos.
The authors have developed a pipeline for analysis of 3D imaging data of neuromasts in the zebrafish posterior lateral line. While most of the individual analysis steps are in themselves not new, as they reference previous studies, they provide a comprehensive semi-automated Python-based workflow for other researchers to follow. Researchers using model systems like the zebrafish, whose transparency facilitates collection of high-resolution 3D imaging data, are faced with significant problem of how to most effectively analyze the images to understand the diversity of cell shape, understand trajectories followed by cells during development, and to compare and analyze changes in these trajectories in experimental and disease conditions. Furthermore, as vast amounts of data related to single cell mRNA expression are collected, there is a need to systematically relate diversity in cell shape and dynamic behavior to diversity in cell type as defined by expression profile. This study lays out step by step how 3D images of individual cells in deposited neuromasts were analyzed by segmenting nuclei and associated cells from confocal images of whole neuromasts. Furthermore, it describes how spherical harmonics (SH) was used to generate a list of 2178 coefficients that describe each cellÂ’s shape and how principal component analysis (PCA) was used to reduce dimensionality to facilitate analysis of cell shape variation within and between cells in the neuromasts. Furthermore, this reduced dimensionality was in most cases adequate to reconstruct the original cell shape.
This approach was independently done for cell and nuclear shape.

© 2024. Published by The Company of Biologists under the terms of the Creative Commons Attribution License (https://creativecommons.org/licenses/by/4.0/).

The efficacy with which reduced dimensionality was adequate to help reconstruct cell shape revealed an important limitation of the spherical harmonics approach and the authors found that cells with concave surfaces were particularly hard to represent. This led to exclusion of 16% of the support cell population from subsequent analyses. Nuclei on the other hand has less variation in shape and their shapes could be reconstructed with just 4 dimensions, as opposed to 8 for cell shape, and no nuclei had to be excluded from.
Unsupervised clustering of cells in cell and nuclear shape was used to identify groups of cells with similar shape and locations. This analysis not only revealed distinctions between central hair cells and surrounding support cells, but also diversity within the support cells, some most prominent in specific quadrants of the neuromast. The authors go on to use previously created transgenic lines to show cell fate markers of support cell subpopulations are associated with cell shape phenotypes. Finally, they describe a new transgenic line in which fluorescent protein coding sequence is inserted in atoh1a in a manner that disrupts its function. By comparing heterozygous and homozygous fish carrying this mutant transgene the authors can show, perhaps predictably, that in the absence of atoh1a function cells with hair cell morphology are lost and change in the fate of these cells increases cells with support cell morphology. This line will contribute to future studies of atoh1a function and to studies related to gene regulatory networks that determine its expression.
Together these studies layout a template workflow for shape analysis and illustrates how such analysis can be used to recognize groups of cells with shared but distinct morphology and how the size of such groups changes following specific manipulations. The study also describes limitations of this approach for cells with concave surfaces. Also, the fact that correlation of cell shape to transgene expression can be confounded by the time it takes for fluorescent protein maturation and the persistence of fluorescent expression in cells that may no longer express genes that determine fluorescent gene expression. The primary value of this study is in defining a workflow and strategy for shape analysis that is expected to have broad application.

Comments for the author

I had no significant suggestions for improvement of the paper. It is well written the figures are easy to follow and the data shown supports the conclusions of the paper.

First revision

Author response to reviewers' comments

We thank the reviewers' careful consideration of our work. Below we address the raised concerns.

Reviewer 1 Comments for the Author:

One things that I am really curious about is why the authors did not trace the same cell over time to test whether a specific cell can indeed be recognised as such at any particular moment?

We agree that it would be interesting to perform time lapse experiments to understand how neuromast cell shapes change over time. However, due to the time duration needed to acquire images at sufficiently high spatial resolution and the large amount of cells needed to perform meaningful clustering analysis, we decided for this work to focus on gathering a representative set of cell shapes from multiple neuromasts at one point in time.

Also, why the authors did not generate a clone to mark some isolated cells (say, in red) and segment them, the same cells but separately, using the green and the red membrane markers. This is to understand better how limiting or confounding the assessment of cell shape may be when looking at a single marker.

It is likely that the use of different markers will result in the delineation of somewhat different cell shapes although we did not examine this specifically. However, as long as one is consistent in the use of a marker and segmentation method, any effects would be the same across all cells analyzed

© 2024. Published by The Company of Biologists under the terms of the Creative Commons Attribution License (https://creativecommons.org/licenses/by/4.0/).

and thus would not invalidate comparisons of shapes between those cells. We now include some discussion of this point in the Limitations section of the discussion

pp3. second paragraphs, it is unclear what "the method", stated twice, refers to. SH or PCA?

We thank the reviewer for pointing out this ambiguity and have changed the wording in this paragraph to be more specific about the method being referred to.

Third paragraph, why do authors call SCs "glia like"? I saw this being done before (Zhang et al., 2018), but I think that it is a mistake because it will confuse the non-expert.

We deleted “glia like” as suggested.

The authors rely exclusively on the Tg(cldnb:LY-EGFP) transgenic line for cell-shape acquisition. I wonder whether possible heterogeneity in the expression level of this transgene across the epithelium may impact segmentation and by extension cell-shape classification.

We did observe some heterogeneity in transgene expression, mainly that expression was lower in the center of the neuromast where the hair cells were located. However, the cell boundaries of the hair cells were still visible. This was mitigated by training a U-Net model to detect cell boundaries. When applying watershed segmentation, we found that the boundary predictions from this model had more consistent values throughout the neuromast, providing a better starting point than raw GFP signal. A similar method was employed to segment cells with membrane labeling in Chen et al. 2020.

pp4. second paragraph "...good quality segmentations for most cells". How was quality assessed to call it good?

The segmentations underwent manual proofreading and correction in 3D by a domain expert, who corrected the labels by hand over multiple rounds until they considered them to be accurate. This differs from fully automated segmentation pipelines in which the quality of segmentation is usually assessed quantitatively using a set of “ground truths” to calculate metrics such as intersection over union and F1 score to assess segmentation quality. In our segmentation pipeline, the quality of segmentation was assessed qualitatively for all neuromasts, rather than only being demonstrated on a subset, and any cells deemed to be low quality were manually selected and excluded. We suggest that these segmentations are essentially 3D ground truths and could be used to develop more fully automated segmentation pipelines in the future. We include discussion of this point in the Limitations section of the discussion

pp5. I do not understand how CSM2 differentiates among SCs, which are less variant that SCs from HCs.

As can be seen in Figure 2 CSM2 scores varied across support cells. SCs at the periphery tended to have lower CSM2 scores than those at the center of the neuromast. Although not as sharp/discrete as the shape variation between SCs as a whole and HCs, the pattern of CSM2 scores suggest that support cells continuously vary in shape as one goes from the center of the neuromast to the periphery, and SCs known to have distinct identities depending on their distance from the center of the neuromast.

pp8. The authros state that "Taken together, these results demonstrate that cells with distinct identities, as indicated by sost:NLS-Eos and sfrp1a:NLS-Eos expression, can be classified by distinct cell shape characteristics." However, this may be indirect and a simple consequence of localization in the neuromast rather than identity.

We acknowledge the reviewer’s point that it is difficult to decouple the effects of cell location and identity on shape, since the locations and identities of neuromast cells are highly associated with each other. To reflect the likely interdependence between these factors, we have amended this

© 2024. Published by The Company of Biologists under the terms of the Creative Commons Attribution License (https://creativecommons.org/licenses/by/4.0/).

statement to read “Taken together, these results demonstrate that cells with distinct identities **and localization**, as indicated by sost:NLS-Eos and sfrp1a:NLS-Eos expression, can be classified by distinct cell shape characteristics."

Reviewer 2 Comments for the Author:

Reviewer 2 had no concerns to address.

Second decision letter

MS ID#: DEVELOP/2023/202251

MS TITLE: Spherical Harmonics Analysis Reveals Cell Shape-Fate Relationships in Zebrafish Lateral Line Neuromasts

AUTHORS: Madeleine N Hewitt, Ivan Cruz, and David W Raible
ARTICLE TYPE: Research Article

I am happy to tell you that your manuscript has been accepted for publication in Development, pending our standard ethics checks. The referee reports on this version are appended below.

Reviewer 1

Advance summary and potential significance to field

This work analyses cell shape in neuromasts of the zebrafish and provides a tool to classify cells quantitatively using morphological features

Comments for the author

The authors answered my questions

Reviewer 2

Advance summary and potential significance to field

I have no comments to add following the original review.

Comments for the author

I have no significant additional suggestions following the initial review

© 2024. Published by The Company of Biologists under the terms of the Creative Commons Attribution License (https://creativecommons.org/licenses/by/4.0/).
